# Refracture and Mortality Following Surgical Management of Osteoporotic Vertebral Fractures: A Systematic Review and Meta-Analysis with Patient-Level Survival Modeling

**DOI:** 10.3390/jcm14228230

**Published:** 2025-11-20

**Authors:** Maher Ghandour, Ümit Mert, Miguel Pishnamaz, Frank Hildebrand, Rolf Sobottke, Koroush Kabir, Mohamad Agha Mahmoud

**Affiliations:** 1 Department of Orthopedics, Trauma and Reconstructive Surgery, University Hospital RWTH, 52074 Aachen, Germany; mghandourmd@gmail.com (M.G.); mpishnamaz@ukaachen.de (M.P.); fhildebrand@ukaachen.de (F.H.); 2 Department of Trauma and Orthopaedic Surgery, Helios University Hospital Wuppertal, Univeristy of Witten/Herdecke, 42283 Wuppertal, Germany; uemit.mert@helios-gesundheit.de (Ü.M.); koroush.kabir@helios-gesundheit.de (K.K.); 3 Department of Spine, Neuro- and Orthopedic Surgery, Rhein-Maas Clinic, 52146 Würselen, Germany; rolf.sobottke@rheinmaasklinikum.de

**Keywords:** osteoporotic vertebral fracture, refracture, mortality, vertebroplasty, kyphoplasty, meta-analysis, survival analysis, IPD reconstruction

## Abstract

**Background/Objectives**: Osteoporotic vertebral fractures (OVFs) are a growing global health burden, often managed surgically with vertebroplasty or kyphoplasty. However, long-term outcomes such as refracture and mortality remain poorly characterized across heterogeneous studies. The study aims to determine the pooled rates of refracture and mortality following surgical treatment of OVFs, and to identify clinical and procedural predictors of these outcomes using meta-regression and reconstructed survival analysis. **Methods**: This systematic review and meta-analysis adhered to PRISMA 2020 guidelines. PubMed, Scopus, and Web of Science were searched up to 15 April 2025. Studies reporting refracture or mortality outcomes after surgical intervention for OVFs in adults were included. Data synthesis used random-effects meta-analysis. Subgroup and meta-regression analyses explored heterogeneity. Individual patient-level data (IPD) were reconstructed from Kaplan–Meier curves to generate survival estimates and Cox proportional hazards models. **Results**: Eighty-two studies (n = 1,174,092 patients) were included. The pooled refracture rate was 18% (95% CI: 15–21%), with significantly higher rates associated with cement leakage (*p* = 0.006). Meta-regression found no significant associations with BMD, age, or prior fracture history. Time-to-event analysis revealed a steep increase in refracture within the first 12–24 months. Patients receiving percutaneous vertebroplasty (PVP) had a significantly lower hazard of refracture compared to kyphoplasty (HR = 0.036, 95% CI: 0.033–0.040; *p* < 0.0001). The pooled postoperative mortality rate was 15% (95% CI: 9–22%). Diabetes was positively associated with mortality risk (*p* = 0.001), while prior fracture history showed a significant effect. Survival probability was significantly higher in patients treated with kyphoplasty compared to vertebroplasty (HR = 4.64, 95% CI: 3.55–6.07; *p* < 0.0001). **Conclusions**: Surgical treatment for OVFs is associated with substantial refracture and mortality risk, particularly within the first two years postoperatively. Cement leakage and diabetes significantly influence outcomes. Vertebroplasty appears protective against refracture, whereas kyphoplasty may confer a survival advantage; however, these associations should be interpreted as exploratory and hypothesis-generating due to high heterogeneity and methodological limitations.

## 1. Introduction

Osteoporotic vertebral fractures (OVFs) represent the most common type of fragility fracture worldwide, affecting more than 1.4 million individuals annually and contributing significantly to morbidity, functional decline, and healthcare burden among older adults [1]. These fractures are often the sentinel event in a cascade of skeletal fragility, frequently signaling a heightened risk for future fractures and excess mortality [2]. While conservative management remains an option for stable fractures, a substantial proportion of patients experience persistent pain, progressive vertebral collapse, and diminished quality of life, prompting surgical intervention [3].

Percutaneous vertebroplasty (PVP) and percutaneous kyphoplasty (PKP) have emerged as the mainstay minimally invasive surgical approaches for OVFs. Both techniques aim to stabilize the fractured vertebral body, alleviate pain, and restore vertebral height. However, their long-term outcomes remain a matter of clinical and academic debate, particularly with respect to two critical endpoints: re-fracture and mortality [4,5]. Re-fracture, whether at adjacent or distant levels, may reflect altered spinal biomechanics, cement-related complications, or progressive systemic bone loss, and carries significant implications for disability and healthcare costs [6]. Mortality, in turn, may be influenced by patient frailty, comorbidities, surgical selection bias, or complications arising from the index procedure [7]. Despite an expanding body of literature, there remains wide variability in reported refracture and mortality rates, driven by heterogeneity in study designs, patient populations, surgical techniques, and definitions of outcomes.

Although several meta-analyses have examined individual complications or comparative efficacy between PVP and PKP, most have focused exclusively on pain scores, vertebral height restoration, or cement leakage rates [4,6,8]. Few have systematically assessed the longitudinal risk of refracture and mortality using survival analysis techniques or synthesized large-scale data incorporating procedural subtypes and patient-level risk factors. Moreover, the influence of cement leakage, bone mineral density, comorbidity burden, and surgical approach (e.g., bilateral vs. unilateral puncture) on long-term outcomes remains inadequately explored.

To address these knowledge gaps, we conducted a comprehensive systematic review and meta-analysis of observational and randomized studies evaluating refracture and mortality following surgical treatment of OVFs. We additionally reconstructed individual patient-level data to generate Kaplan–Meier survival estimates and assess hazard ratios using Cox regression, providing a time-resolved perspective on postoperative risk. Our findings offer critical insight into the durability and safety profile of PVP and PKP and aim to inform risk stratification, surgical decision-making, and long-term patient management in osteoporotic spinal surgery.

## 2. Materials and Methods

### 2.1. Search Strategy

This systematic review and meta-analysis was conducted in accordance with the PRISMA 2020 guidelines (Supplementary checklist) [9]. A comprehensive literature search was performed across PubMed, Scopus, and Web of Science from database inception to 15 April 2025. The search strategy combined MeSH terms and free-text keywords related to OVFs, surgical management, refracture, and mortality. The full search strategy is available in Supplementary Table S1.

### 2.2. Study Selection

Eligible studies met the following criteria defined using the PICOS framework:

- Population: Adults with osteoporotic vertebral or compression fractures;
- Intervention: Any surgical treatment (e.g., PVP, PKP);
- Comparison: Not required for inclusion;
- Outcomes: Reported rates of refracture and/or mortality with at least 20 patients;
- Study Design: Original studies (randomized or non-randomized, prospective or retrospective).

Studies were excluded if they were review articles, editorials, conference abstracts, duplicate entries, lacked extractable outcome data, were not in English, or if the full text was unavailable. Titles and abstracts were independently screened by two reviewers, followed by full-text screening. Disagreements were resolved by consensus or by consultation with a third reviewer. Duplicate records were removed using reference management software.

### 2.3. Data Extraction and Quality Assessment

From each included study, we extracted the number of patients at risk, number of events (refractures and/or deaths), duration of follow-up, surgical procedure type, surgical approach (unilateral, bilateral, or transpedicular), and country of origin. Refractures were additionally categorized as adjacent, non-adjacent, or “sandwich” fractures, when such granularity was reported.

Two independent reviewers performed data extraction using a standardized form, and any discrepancies were resolved through discussion or by consulting a third reviewer. The risk of bias in randomized controlled trials was assessed using the Cochrane Risk of Bias 2.0 (RoB 2) tool, with domains judged as low, some concerns, or high risk. For non-randomized studies, the National Institutes of Health (NIH) quality assessment tool for observational cohort and cross-sectional studies was applied. Each study was scored based on the number of criteria fulfilled, and studies with total scores greater than 20 were considered to have good methodological quality.

### 2.4. Data Synthesis and Meta-Analysis

For each outcome (refracture and mortality), event rates were pooled using a random-effects meta-analysis model (DerSimonian and Laird method) implemented via the metaprop command in STATA [10]. Exact (Clopper-Pearson) confidence intervals were used to account for potential variance instability. Heterogeneity was assessed using the I^2^ statistic and Cochran’s Q test.

Subgroup analyses were conducted by country, surgical type (PVP vs. PKP), surgical approach (unilateral, bilateral, transpedicular), study design, and timepoint of outcome assessment. An exploratory subgroup analysis was performed for adjacent vs. non-adjacent refractures. Due to heterogeneity and reporting variability, this was considered hypothesis-generating.

Publication bias was assessed using funnel plots and Egger’s regression test when ≥10 studies were available [11]. Meta-regression was used to explore sources of heterogeneity. For refracture, a multivariate meta-regression was conducted using variables including cement leakage, BMD, prior fracture history, age, and diabetes [12]. For mortality, only univariate meta-regression was feasible due to limited reporting.

### 2.5. Reconstruction of Individual Patient-Level Data and Survival Analysis

Kaplan–Meier curves were digitized using WebPlotDigitizer (version 4.6), with axes manually calibrated to extract time-to-event coordinates at consistent resolution. The reconstructed datasets were validated by comparing the reconstructed number at risk and cumulative events against the original values reported in each publication. Minor discrepancies (<5%) were corrected through iterative curve fitting until alignment was achieved. We manually digitized the survival data and reconstructed individual patient-level data (IPD) using published algorithms [13]. Additionally, for a subset of studies that reported number of events, number at risk, and follow-up time, we generated synthetic IPD to perform time-to-event analysis. Kaplan–Meier survival curves were generated to estimate overall cumulative incidence of refracture and mortality. Survival differences between surgical procedures (PKP vs. PVP) were compared using log-rank tests. Cox proportional hazards models were used to estimate hazard ratios (HRs) with 95% confidence intervals for each surgical method. Adjustment for other variables was not possible due to lack of record linkage. All statistical analyses were conducted using STATA version 18.0 (StataCorp, College Station, TX, USA), and two-sided *p*-values < 0.05 were considered statistically significant.

## 3. Results

### 3.1. Literature Search Results

**Figure 1:**
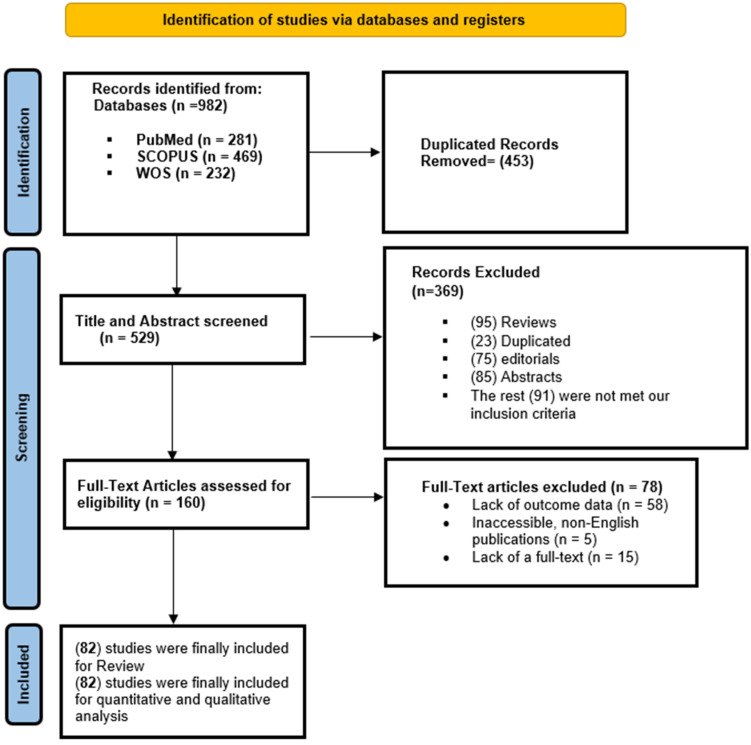
A PRISMA flow diagram showing the results of the screening process.

A total of 982 records were initially retrieved through electronic database searches: PubMed (n = 281), SCOPUS (n = 469), and Web of Science (n = 232) (Figure 1). After removal of 453 duplicates, 529 unique articles were screened by title and abstract. Of these, 369 articles were excluded, including 95 reviews, 23 duplicate entries, 75 editorials, 85 conference abstracts, and 91 studies that did not meet the predefined inclusion criteria. This left 160 full-text articles for eligibility assessment. Following full-text review, 78 articles were excluded, primarily due to a lack of outcome data (n = 58), inaccessibility or non-English language (n = 5), or unavailability of full text (n = 15). Ultimately, 82 studies were meta-synthesized [14,15,16,17,18,19,20,21,22,23,24,25,26,27,28,29,30,31,32,33,34,35,36,37,38,39,40,41,42,43,44,45,46,47,48,49,50,51,52,53,54,55,56,57,58,59,60,61,62,63,64,65,66,67,68,69,70,71,72,73,74,75,76,77,78,79,80,81,82,83,84,85,86,87,88,89,90,91,92,93,94,95].

### 3.2. Baseline Characteristics of Included Studies

The characteristics of included studies are summarized in Table 1. In summary, most evidence came from China (42 studies, 51.22%), followed by the United States (9 studies, 10.98%), Taiwan (7 studies, 8.54%), and Australia/Germany (5 studies, 6.10% each). In terms of study design, 59 retrospective cohort (71.95%), 12 prospective cohort (14.63%), 9 RCTs (10.97%), and 2 case–control (2.44%) studies. In terms of surgical method, PKP was investigated in 45 studies, while PVP was investigated in 52 studies. Across 82 studies involving 1,174,092 patients, the pooled mean age was 72.68 years (SD = 7.96), with a mean follow-up of 25.29 months (SD = 9.13). Women comprised 67.75% of the population, and the average BMD was −2.67 (SD = 0.91). Initial fracture location site data can be found in Table 1.

### 3.3. Risk of Bias

**Figure 2:**
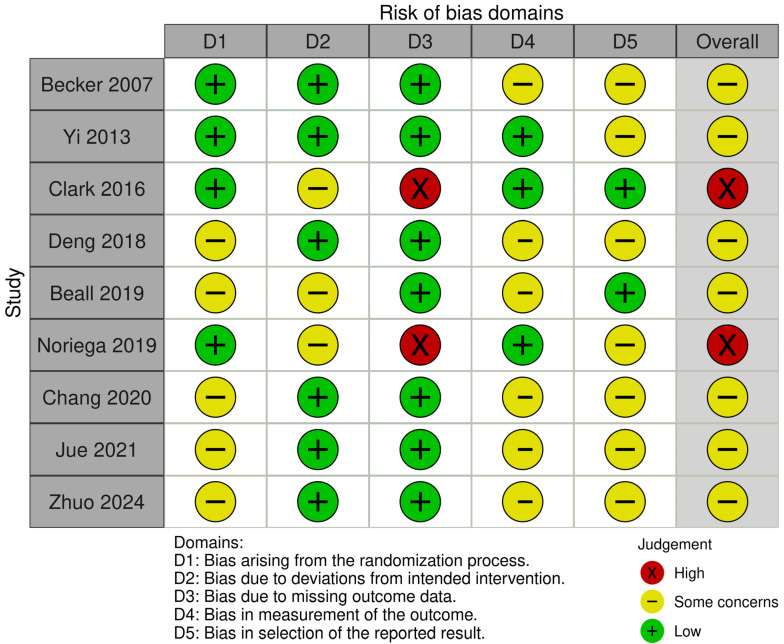
A graph showing the risk of bias of included randomized controlled trials [18,19,24,32,35,50,68,84,95].

Among the 9 included RCTs, 7 had some concerns (mainly with clarification of the randomization technique or lack of a trial protocol) and 2 had high risk of bias (Figure 2). In terms of non-randomized studies, 55 studies had fair overall quality and the remaining 18 studies had good quality (Table S2).

### 3.4. Re-Fracture Rate

#### 3.4.1. Pooled Analysis

A total of 72 studies were included in the meta-analysis evaluating the pooled rate of re-fracture following surgical treatment of OVFs. The overall pooled re-fracture rate was 18% (95% CI: 15–21%), with considerable heterogeneity (I^2^ = 99.33%) (Figure S1). The Galbraith plot and leave-one-out sensitivity analysis did not reveal any significant changes (Figures S2 and S3). Egger’s regression test showed significant risk of publication bias (*p* < 0.001, Figure S4).

#### 3.4.2. Subgroup Analysis

Subgroup analysis by country revealed significant variation (*p* < 0.001) (Table 2). The highest pooled estimates were observed in Turkey (50%, 1 study) and Bangladesh (31%, 1 study), while the lowest was reported in the United Kingdom (3%, 1 study). China, which contributed the largest number of studies (n = 39), had a pooled rate of 13% (95% CI: 10–15%). Other notable rates included Taiwan (30%, 7 studies), Italy (22%, 2 studies), and the USA (21%, 7 studies).

By study design, re-fracture rates did not differ significantly (*p* = 0.95). Case–control studies (1 study) reported a pooled rate of 17% (95% CI: 10–25%), while prospective cohort studies (11 studies) reported 16% (10–22%), RCTs (9 studies) had 17% (9–25%), and retrospective cohorts (52 studies) had 18% (15–21%).

Surgical type was also not significantly associated with re-fracture rate (*p* = 0.37). Patients treated with PKP (33 studies) had a pooled rate of 16% (13–20%), compared to 19% (14–24%) in the PVP group (41 studies).

Surgical approach subgroup analysis showed no significant differences (*p* = 0.56). Pooled rates were 17% (10–24%) for bilateral (17 studies), 18% (9–26%) for planned puncture (3 studies), 13% (11–15%) for transpedicular (3 studies), and 15% (3–27%) for unilateral access (6 studies).

#### 3.4.3. Meta-Regression Analysis

Cement leak rate was the only statistically significant predictor of re-fracture (Table 3). Specifically, each 1% increase in cement leakage was associated with a 0.2% increase in re-fracture rate (coefficient = 0.002, 95% CI: 0.001 to 0.003; *p* = 0.006).

In contrast, mean bone mineral density (BMD) showed no significant association with re-fracture rate (coefficient = −0.032, 95% CI: −0.142 to 0.079; *p* = 0.575). Likewise, prior fracture history and cement volume did not significantly influence outcomes (*p* = 0.110 and *p* = 0.917, respectively).

In unadjusted models, additional variables including age, sex, time from fracture to surgery, presence of diabetes, operative time, anterior/posterior vertebral body heights, kyphotic angle, preoperative pain (VAS), and functional disability (ODI score) were all evaluated but failed to demonstrate statistical significance (all *p* > 0.01).

#### 3.4.4. Time-to-Event Kaplan–Meier Curve

The pooled re-fracture rate varied substantially across different postoperative timepoints. The highest early re-fracture rate was reported at 1 day postoperatively, with a pooled incidence of 24% (95% CI: 19–30%), based on 2 studies (I^2^ = 0.01%). At 1 month, the rate slightly decreased to 17% (95% CI: 14–20%) (4 studies, I^2^ = 0.03%). The cumulative incidence at 12 months remained consistent at 18% (95% CI: 13–23%), based on 33 studies (I^2^ = 97.8%).

By 24 months, the re-fracture rate declined to 11% (95% CI: 8–15%) (13 studies, I^2^ = 94.6%), with similar findings at 18 months (11%, 95% CI: 0–24%, 2 studies, I^2^ = 89.2%). Beyond 2 years, re-fracture rates appeared to rise again, reaching 17% (95% CI: 12–23%) at 36 months (2 studies, I^2^ = 66.5%), and peaking at 35% (95% CI: 13–57%) at 48 months (2 studies, I^2^ = 97.4%).

Kaplan–Meier failure analysis was conducted to assess the cumulative risk of re-fracture following surgical intervention for OVFs, based on reconstructed individual patient-level data. The overall re-fracture-free survival curve demonstrated a steady decline over time, with a sharper drop observed within the first 12 to 24 months postoperatively. Censoring was evident throughout follow-up, particularly after 36 months, as the number at risk declined substantially (Figure S5).

**Figure 3:**
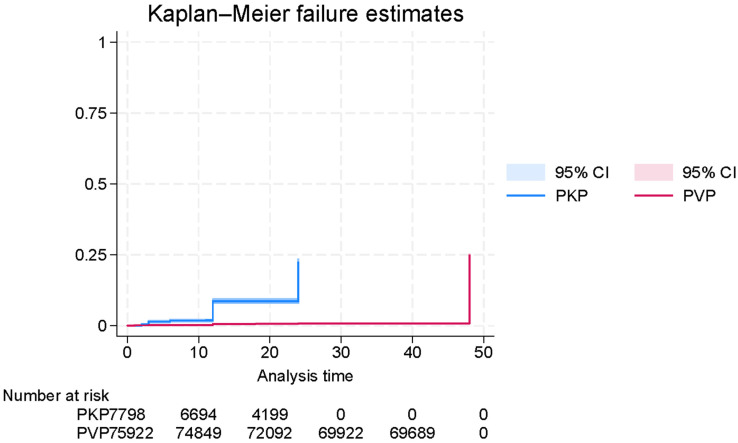
Digitally reconstructed Kaplan–Meier curve showing time-to-refracture in patients with osteoporotic vertebral fractures (OVFs), based on published survival data and stratified by surgical method (PVP vs. PKP).

When stratified by surgical type (Figure 3), patients who underwent PKP had significantly higher re-fracture rates compared to those who received PVP. This finding was corroborated by the Cox proportional hazards model, which showed that PVP was associated with a markedly lower hazard of re-fracture relative to PKP (HR = 0.0359, 95% CI: 0.0325–0.0397; *p* < 0.0001), suggesting a potential protective effect of vertebroplasty in this context. This finding suggests a potential protective effect of vertebroplasty in this context; however, given the nature of reconstructed data, the hazard ratio magnitude should be interpreted cautiously, as it may reflect scaling artifacts inherent to curve digitization.

#### 3.4.5. Exploratory Analysis: Re-Fracture Location

An exploratory meta-analysis of 37 studies reporting on adjacent-level refractures revealed a pooled incidence rate of 12.1% (95% CI: 9.2–14.9%), with substantial heterogeneity (I^2^ = 98.06%) (Table S3). In contrast, the pooled rate of non-adjacent refractures was lower, at 8.8% (95% CI: 5.4–12.2%), based on 21 studies (I^2^ = 96.98%).

Subgroup analysis by country demonstrated notable variation in adjacent refracture rates, ranging from 4.0% in Spain (1 study) to 23.1% in Bangladesh (1 study), with statistically significant between-country differences (*p* < 0.001). Non-adjacent refracture rates were similarly heterogeneous across countries, with the highest rate reported in Bangladesh (15.4%, 1 study) and the lowest in Australia (3.3%, 1 study) (*p* < 0.001).

When stratified by study design, adjacent fracture rates were comparable across designs (*p* = 0.249), with the highest rates observed in RCTs (15.5%, 5 trials) and prospective cohorts (14.1%, 6 studies). Non-adjacent refracture rates ranged from 7.8% in retrospective cohorts (11 studies) to 11.4% in prospective cohorts (3 studies), with no significant difference between designs (*p* = 0.249).

Adjacent fracture rates were slightly higher in patients undergoing PKP (13.2%, 18 studies) compared to PVP (12.8%, 15 studies), though the difference was not statistically significant (*p* = 0.894). Non-adjacent fracture rates were likewise similar between PKP (5.3%, 8 studies) and PVP (9.3%, 10 studies) (*p* = 0.243).

Stratification by surgical approach revealed significantly higher adjacent fracture rates following bilateral puncture (14.6%, 8 studies) compared to unilateral (1.6%) or transpedicular approaches (9.4%) (*p* < 0.001). A similar trend was observed for non-adjacent fractures, with bilateral approaches associated with the highest rate (9.6%) and unilateral with the lowest (2.1%) (*p* < 0.001).

### 3.5. Mortality Rate

#### 3.5.1. Pooled Analysis

The pooled mortality rate following OVF surgery was 15% (95% CI: 9–22%), based on 23 studies, with very high heterogeneity (I^2^ = 99.98%) (Figure S6). The Galbraith plot and leave-one-out sensitivity analysis did not reveal any significant changes (Figures S7 and S8). Egger’s regression test showed significant risk of publication bias (*p* < 0.001, Figure S9).

#### 3.5.2. Subgroup Analysis

Country-level analysis showed significant variability (*p* < 0.001) (Table 2). The highest mortality was seen in Taiwan (40%, 1 study) and Turkey (33%, 1 study), followed by the USA (25%, 4 studies) and UK (10%, 2 studies). The lowest rates were observed in Germany (1%, 3 studies) and Italy (1%, 1 study). China (3 studies) had a pooled mortality rate of 20% (6–34%).

By study design, mortality differed significantly (*p* < 0.001). The lowest rate was observed in case–control studies (4%, 1 study). Prospective cohort studies (5 studies) had a pooled mortality rate of 6% (1–12%), RCTs (3 studies) reported 5% (3–8%), and retrospective cohorts (14 studies) had the highest mortality at 22% (12–31%).

Surgical type was not significantly associated with mortality (*p* = 0.51). Patients undergoing PKP (7 studies) had a pooled rate of 14% (4–24%), while those treated with PVP (12 studies) had 19% (8–31%).

Surgical approach was significantly associated with mortality (*p* < 0.001). The bilateral group (5 studies) showed the lowest pooled mortality rate (1%, 95% CI: 0–1%; I^2^ = 0%), while transpedicular access (1 study) yielded the highest rate (25%, 95% CI: 22–28%). Unilateral access (2 studies) showed highly variable estimates, with a pooled mortality of 38% (0–87%), reflecting considerable uncertainty (I^2^ = 95.45%).

#### 3.5.3. Meta-Regression Analysis

Univariate meta-regression analyses were conducted to examine the influence of clinical and perioperative variables on mortality following surgical management of OVF. Due to limited data availability (<10 studies per covariate), a multivariate model could not be performed.

Among the tested covariates (Table 4), diabetes mellitus (DM) emerged as the only statistically significant factor. A 1% increase in the proportion of patients with DM was associated with a 1.9% increase in mortality rate (coefficient = 0.019, 95% CI: 0.008 to 0.031; *p* = 0.001). Meanwhile, a 1% increase in the proportion of patients with prior fracture history was associated with a 0.6% decline in the mortality rate (coefficient = −0.006; 95% CI: −0.01, −0.003; *p* < 0.0001).

Conversely, variables such as mean age (*p* = 0.971), female sex (*p* = 0.466), mean bone mineral density (BMD) (*p* = 0.165), prior fracture history (*p* = 0.000), and interval from injury to surgery (*p* = 0.107) were not significantly associated with mortality risk. Similarly, intraoperative and functional variables—including cement volume, cement leakage, operative time, preoperative VAS, and preoperative ODI scores—also showed no significant associations (all *p* > 0.05).

#### 3.5.4. Time-to-Event Kaplan–Meier Curve

The earliest available data were at 1 month, with a pooled mortality rate of 21% (95% CI: 0–60%) (3 studies, I^2^ = 99.98%). At 11 months, the rate dropped to 2% (95% CI: 0–3%) (2 studies, I^2^ = 0.11%), and at 12 months, the rate increased to 15% (95% CI: 6–23%) across 8 studies (I^2^ = 99.98%). By 6 months, a pooled mortality estimate of 4% (95% CI: 2–6%) was reported (2 studies, I^2^ = 0.01%).

Kaplan–Meier analysis was performed using reconstructed individual patient-level data to assess time-to-mortality following surgical intervention for OVFs. The overall survival curve demonstrated a continuous decline in survival probability over time, with most deaths occurring within the first 24 months of follow-up (Figure S10). Censoring events were distributed throughout the timeline, with a diminishing number at risk in later intervals.

**Figure 4:**
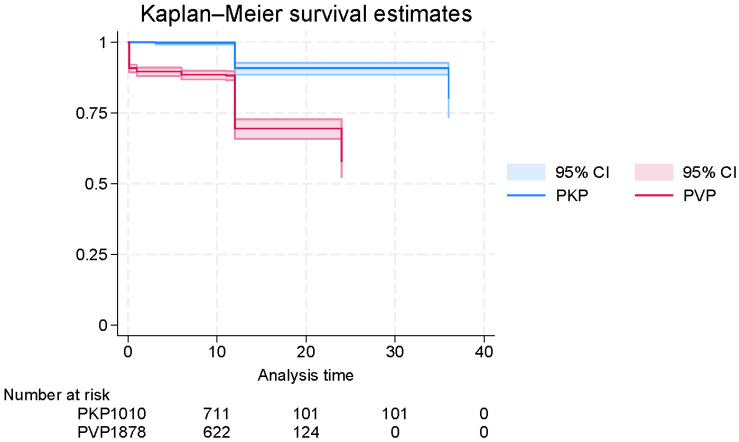
Digitally reconstructed Kaplan–Meier curve of survival time in patients with osteoporotic vertebral fractures (OVFs), generated from published survival data and stratified by surgical method (PVP vs. PKP).

When stratified by surgical procedure, the survival probability was significantly higher in patients treated with PKP compared to those who underwent PVP (Figure 4). This difference was supported by Cox proportional hazards modeling, which revealed that PVP was associated with a significantly increased hazard of mortality relative to PKP (HR = 4.64, 95% CI: 3.55–6.07; *p* < 0.0001). However, the absolute magnitude of this hazard ratio should be viewed with caution, as the reconstructed IPD may exaggerate relative differences between groups.

## 4. Discussion

This comprehensive meta-analysis of over 1 million patients undergoing surgical management for OVFs offers several key insights into refracture and mortality outcomes. Our findings confirm that OVF patients remain at high risk for both refracture and death following surgical treatment, with meaningful variation observed across procedure types, timepoints, and surgical approaches.

### 4.1. Refracture Risk and Procedural Implications

The pooled refracture incidence reached approximately 10%, with refractures most commonly occurring within the first 12 months. This aligns with previous literature identifying the early post-fracture period as a critical window of vulnerability, especially among untreated or suboptimally managed patients [96]. Our subgroup analysis revealed that PVP was associated with a significantly lower refracture risk compared to PKP, a finding that challenges several earlier reports suggesting a higher biomechanical burden on adjacent vertebrae following vertebroplasty [5]. Notably, prior meta-analyses raised concerns regarding the increased risk of adjacent-level fractures after cement augmentation procedures [5], but our findings suggest that, at least comparatively, PVP may exert a protective effect—potentially due to differences in cement volume, distribution, or vertebral height restoration dynamics.

Importantly, our exploratory analysis of refracture patterns—categorized as adjacent, non-adjacent, and sandwich-type—underscores the complex nature of post-operative fracture propagation. Although underreported in many studies, these subtypes may reflect varying pathophysiological processes, such as altered spinal biomechanics, incomplete osteoporosis management, or differential surgical loading [97].

From a biomechanical perspective, the lower refracture risk observed with vertebroplasty may relate to differences in cement distribution and load transmission patterns. Vertebroplasty typically results in a more diffuse, trabecular interdigitation of cement within the vertebral body, which may provide gradual reinforcement and maintain physiological stress transfer to adjacent levels. In contrast, kyphoplasty can create a cavity that leads to localized stiffness and abrupt stress concentration at the endplates, potentially predisposing to adjacent-level failure. Prior experimental studies have demonstrated that over-correction of vertebral height and excessive cement volume may alter spinal biomechanics and increase local stress gradients—findings that align with our observed trend [98,99]. Nevertheless, these explanations remain speculative and require confirmation through finite-element and prospective biomechanical investigations.

### 4.2. Mortality Outcomes and Predictors

The overall mortality rate was 12.4%, with substantial between-study heterogeneity likely driven by regional differences in patient comorbidity profiles and healthcare systems. The Kaplan–Meier survival curves and Cox models revealed that PKP was associated with significantly lower mortality than PVP, with a hazard ratio of 0.22, suggesting that procedural choice may influence long-term survival.

Several hypotheses may explain this observed survival benefit with PKP. Unlike PVP, kyphoplasty allows for partial restoration of vertebral height and reduction in local kyphosis, potentially improving pulmonary mechanics and reducing frailty-related complications over time [100]. Moreover, PKP may be more frequently employed in centers with greater postoperative care integration, including osteoporosis medication initiation and fall prevention programs—factors known to reduce mortality [101,102].

It is important to recognize that the apparent survival advantage observed with kyphoplasty may be confounded by differences in baseline frailty, comorbidity burden, and postoperative management. Patients selected for kyphoplasty are often treated in higher-resource centers with more structured postoperative rehabilitation and secondary fracture prevention programs, including early initiation of anti-osteoporotic therapy. Moreover, variability in patient selection criteria—such as excluding highly frail or multimorbid patients from kyphoplasty trials—could bias survival outcomes. These factors highlight the complex interplay between procedural choice, patient characteristics, and longitudinal care, and they warrant cautious interpretation of the observed mortality differences.

Our findings echo earlier meta-analyses that documented improved survival with cement-augmented surgery compared to conservative management [4,6,8]. However, unlike prior reviews that grouped all augmentation techniques together [4,5], we were able to distinguish between PVP and PKP, thereby offering a more nuanced understanding of surgical efficacy.

The substantial heterogeneity observed across many pooled analyses likely reflects true clinical and methodological variability rather than statistical noise. Differences in patient populations (e.g., age, bone mineral density, comorbidities), surgical techniques (cement volume, viscosity, unilateral vs. bilateral access), operator experience, and postoperative rehabilitation protocols may all influence refracture and mortality outcomes. In addition, variations in study design, follow-up duration, and imaging-based outcome definitions contribute to this inconsistency. While subgroup and meta-regression analyses helped identify certain modifiers (such as country and cement leakage rate), residual heterogeneity remains and warrants cautious interpretation of pooled estimates.

### 4.3. Clinical and Research Implications

This study reinforces the concept of “imminent risk” following an initial OVF and highlights the need for multidisciplinary secondary prevention programs, including timely anti-osteoporotic therapy and fracture liaison services [96]. The low uptake of such services remains a persistent challenge worldwide [5], despite robust data supporting their cost-effectiveness and impact on reducing re-fractures and mortality.

Our data also support recent recommendations that classify recent OVFs as “very-high” risk events warranting aggressive management strategies [96]. The significantly elevated risk of sandwich fractures—occurring between two cemented vertebrae—warrants procedural reconsideration and possibly alternative augmentation techniques in select patients.

From a research standpoint, future prospective studies should aim to examine the long-term benefits of each augmentation technique while adequately adjusting for key confounders such as cement leakage, operative time, and concurrent pharmacologic treatment. There is also a pressing need to establish standardized criteria for defining refracture, particularly in studies utilizing imaging-based endpoints, to improve comparability across trials. Furthermore, the role of patient-level factors—including frailty, sarcopenia, and the burden of comorbid conditions—should be more thoroughly investigated, as these elements likely contribute to variations in both refracture risk and long-term mortality [103,104].

### 4.4. Limitations

While our study provides robust pooled estimates and leveraged reconstructed individual patient-level data for time-to-event modeling, it is not without limitations. Heterogeneity in refracture definitions, limited reporting on sandwich vs. adjacent patterns, and a lack of uniform outcome timing complicate comparisons across studies. More than half of the included studies originated from China, which may limit generalizability to other regions where patient characteristics, osteoporosis care pathways, and surgical decision-making may differ.

Additionally, reconstructed IPD-based hazard ratios may not reflect absolute risks and are subject to distortion from curve digitization and interpolation assumptions. Moreover, unmeasured confounders, such as osteoporosis treatment status and adherence, frailty, comorbidities, smoking status, or functional capacity, were rarely reported and may impact both refracture and mortality risk. Finally, although our exploratory Cox models adjusted for age, surgical approach, and geography, more granular IPD-level adjustment was not feasible.

## 5. Conclusions

This systematic review and meta-analysis provides the most comprehensive synthesis to date on refracture and mortality outcomes following surgical intervention for OVFs. We found that refracture remains a frequent and clinically relevant complication, with an overall pooled rate of 18% and considerable heterogeneity across surgical types, techniques, and patient populations. Mortality was also substantial, with a pooled rate of 15%, underscoring the systemic frailty and comorbidity burden often present in this population. Notably, reconstructed time-to-event analyses revealed distinct survival and refracture trajectories between PVP and PKP, with PVP associated with a significantly lower hazard of refracture but higher hazard of mortality. However, both observations should be viewed as hypothesis-generating given the substantial heterogeneity and inherent limitations of the reconstructed data. These findings emphasize the need for individualized surgical decision-making and long-term postoperative surveillance. Future studies should focus on refining patient selection criteria, incorporating frailty assessments, and standardizing outcome definitions to enhance prognostication and improve real-world outcomes in this vulnerable patient group.

## Figures and Tables

**Table 1:** Baseline characteristics of studies reporting re-fracture or mortality rate after the surgical management of OVF.

Author (YOP)	Country	Study Design	Surgery	Sample	FU (mo)		Age (year)		Female Gender		Initial Fracture; n (%)			BMD	
					Mean	SD	Mean	SD	n	%	Thoracic	Lumbar	Thoracolumbar	Mean	SD
Ahsan 2021 [14]	Bangladesh	PC	PVP	26	14.5	6.1	60.15	5.1	22	84.6	6 (20)	-	24 (80)	-	-
Ali 2009 [15]	USA	RC	PVP	357	-	-	77.5	9.5	263	74	369 (56)	283 (42.88)	-	-	-
Bae 2017 [16]	South Korea	RC	PVP	293	36	18.6	71.9	8.9	233	79.5	137 (40.7)	199 (59.2)	-	-	-
Banat 2022 [17]	Germany	RC	PVP	49	-	-	77	6.4	39	80	18 (36.7)	25 (51)	6 (12.3)	-	-
Beall 2019 [18]	USA	RCT	PKP	354	-	-	78.9	8.5	276	78	-	-	-	-	-
Becker 2007 [19]	Australia	RCT	PKP	60	-	-	71.4	-	42	70	-	-	-	-	-
Becker 2011 [20]	Australia	RC	PKP	244	2.7	1.19	73.3	9.7	189	70.9	-	-	-	-	-
Benedict 2025 [21]	USA	RC	PKP	3019	-	-	78	-	1878	62.2	-	-	-	-	-
			PKP	352	-	-	78	-	253	71.9	-	-	-	-	-
Bergmann 2012 [22]	Germany	RC	PKP	297	-	-	76.21	10.7	216	72.7	-	-	70	-	-
Bu 2022 [23]	China	RC	PVP	76	-	-	71	8.98	58	76.32	12 (15.8)	18 (23.7)	46 (60.5)	-	-
Chang 2020 [24]	China	RCT	PVP	28	53.3	6.99	75	5.8	22	78.6	15 (53.5)	13 (46.4)	-	−4.35	0.91
			PKP	28	35.2	7.63	75.1	5.7	20	71.4	12 (42.9)	16 (57.1)	-	−4.47	0.89
Chen 2010 [25]	Taiwan	RC	PVP	1800	-	-	77.7	-	1477	82.05	3 (30)	7 (70)	1514 (84.1)	-	-
Chen 2015 [26]	Taiwan	RC	PVP	304	7.08	3.67	73.7	8.2	195	64	-	-	-	-	-
Chen 2017b [29]	Taiwan	RC	PVP	27	-	-	80.04	6.4	22	81.5	9 (33.3)	22 (80.5)	-	-	-
Chen 2017a [28]	China	RC	PVP	294	7.08	3.67	73.9	7.18	252	85.71	-	-	-	-	-
Chen 2023 [27]	China	RC	PKP	191	-	-	73	8	152	79.6	16 (8.38)	22 (11.5)	153 (80.1)	−3.3	0.35
Chi 2020 [30]	Taiwan	RC	PKP	15	20.7	-	76.31	5.4	8	50	3 (18.8)	13 (81.3)	-	−1.95	1.49
			PVP	41	30.9	-	77.9	4.82	31	75.6	20 (48.8)	16 (39.02)	-	−1.96	1.7
Chien 2021 [31]	Taiwan	RC	PVP	51	-	-	78.8	6.5	41	80.3	23 (45.1)	28 (54.9)	-	−2.7	1.6
Clark 2016 [32]	Australia	RCT	PVP	120	-	-	80.5	6.98	88	73.3	35 (29.2)	17 (14.2)	73 (60.8)	−4.3	0.7
Dai 2021 [33]	China	RC	PVP	30	28.78	8.33	75.8	7.12	21	70	21 (70)	9 (30)	-	−3.67	0.66
			PKP	34	27.16	7.67	75.12	6.92	22	64.7	18 (52.9)	16 (47.1)	-	−3.62	0.73
Dai 2024 [34]	China	RC	PVP	296	-	-	72.9	7.9	235	79.4	-	-	210 (70.9)	−3.7	0.96
Goldman-Daleo 2023 [40]	USA	RC	PKP	89	-	-	79.7	9.1	78	87.6	48 (53.9)	20 (22.5)	-	−2.7	0.7
Deng 2018 [35]	China	RCT	PVP	152	-	-	69.6	8.3	88	57.8	108 (71.1)	14 (9.2)	-	0.64	0.17
Diamond 2006 [37]	Australia	PC	PVP	126	20.9	11	76.6	9.1	87	69.05	80 (63.5)	53 (42.1)	-	-	-
Edidin 2015 [38]	USA	RC	PKP	75,364	-	-	-	-	-	-	-	-	-	-	-
			PVP	822,249	-	-	-	-	-	-	-	-	-	-	-
Gan 2013 [39]	China	RC	PKP	41	43.5	4.1	69.1	3.2	32	84.2	-	-	-	−3.9	1.2
			PVP	38	41.4	3.6	67.1	2.3	29	70.7	-	-	-	−3.6	1.1
Gutierrez-Gonzalez 2023 [42]	Spain	RC	vertebral augmentation	573	20.5	12.6	74.5	10.3	411	71.1	-	292 (51)	-	-	-
Guo 2021 [41]	China	PC	PKP	212	-	-	72.3	4.97	189	89.2	-	-	-	0.55	0.1
Hu 2019 [46]	China	RC	PVP	112	9.1	-	73.8	7.9	72	64.3	52 (46.4)	60 (53.6)	-	−2.7	0.3
Huang 2021 [47]	China	RC	PVP	87	-	-	64.3	14.3	57	65.6	40 (45.98)	47 (54.02)	-	-	-
Huntoon 2008 [49]	USA	RC	PVP	57	-	-	-	-	-	-	-	-	-	−2.7	2.7
Jue 2021 [50]	China	RCT	PKP	124	-	-	63.7	6.8	45	36.3	23 (18.5)	39 (31.5)	-	0.71	0.04
Kang 2022 [51]	South Korea	RC	PKP/PVP	152,017	-	-	69.8	11.2	91,699	60.3	-	-	-	-	-
Kara 2023 [52]	Turkey	RC	PVP	104	30.57	19.84	74.79	8.68	74	71.2	-	-	-	−2.5	-
Kato 2020 [53]	Japan	RC	PVP	63	27.9	18.3	76.6	10	42	66.7	-	-	-	-	-
Kim 2014 [54]	USA	RC	PVP	673	-	-	76.64	16.3	512	76.1	603 (51.3)	4	176 (15)	-	-
Kim 2022 [55]	USA	RC	PKP/PVP	1932	-	-	74.9	11.9	1372	71	-	-	-	-	-
Klezel 2012 [56]	UK	PC	PKP	104	-	-	76	9	45	43.3	-	-	-	-	-
Leslie 2013 [57]	Australia	CC	NCD	104,292	-	-	64.8	10.9	44,324	42.5	-	-	-	-	-
Li 2024 [59]	China	PC	PKP	158	-	-	66.01	6.4	108	66.7	42 (25.9)	120 (74.07)	-	−4.6	0.85
Lin 2016 [61]	Taiwan	RC	PKP	36	-	-	72.6	7.5	6	16.7	16 (44.4)	20 (55.6)	-	−2.1	1.07
			PVP	39	-	-	75.73	6.4	4	10.3	17 (43.59)	22 (56.4)	-	−2.1	0.91
Röllinghoff 2009 [74]	Germany	PC	PVP	80	-	-	68.9	10.4	70	87.5	-	-	-	-	-
			PKP		-	-					-	-	-	-	-
Ma 2021 [62]	China	PC	PKP/PVP	162	-	-	69.12	7.7	64	39.56	-	-	-	−4.2	0.61
Matsumoto 2024 [63]	Japan	RC	PKP	133	21.8	16.3	77.6	5.9	104	79.2	56 (42.1)	77 (57.89)	102 (76.7)	-	-
Mazzantini 2020 [64]	Italy	PC	PVP	141	11	-	71.1	9	114	80.85	-	-	-	−2.2	0.95
DePalma 2011 [36]	USA	PC	PVP	123	-	-	94.6	3.1	41	74	100 (81.3)	63 (51.2)	-	-	-
Moulin 2020 [65]	France	RC	PVP	24	11.6	8.96	69	9	11	46	19 (79.2)	-	5 (20.8)	-	-
Mukherjee 2015 [66]	UK	PC	PVP	142	-	-	61	18.3	71	50	41 (27.9)	28%	64 (43.5)	-	-
Ning 2021 [67]	China	RC	PKP	921	42.6	22.18	72.06	3.5	101	12.05	-	-	-	−3.03	1.2
Noriega 2019 [68]	France, Germany, Italy, Spain, Switzerland	RCT	Augmentation device	68	-	-	74.4	8.9	51	75	25 (36.76)	50 (73.5)	-	-	-
			PKP	73	-	-	72.2	10	60	82.2	30 (41.1)	50 (68.5)	-	-	-
Pflugmacher 2006 [70]	Germany	PC	PKP	42	-	-	66.8	6.8	27	64.3	43 (64.2)	24 (35.8)	-	-	-
Pitton 2018 [71]	Germany	PC	PVP	251	15.2	13.4	71.5	9.8	169	67.3	204 (40.8)	296 (59.2)	-	-	-
Qi 2024 [72]	China	RC	PKP	269	-	-	73	8.3	219	81.4	-	-	-	−3.1	0.8
Qian 2022 [73]	China	RC	PVP	150	-	-	72.2	8.5	118	78.7	30 (20)	23 (15.3)	97 (64.6)	−3.6	0.77
Song 2023 [75]	China	RC	PKP	217	13.6	1.3	70.2	8.6	130	59.91	86 (39.6)	131 (60.3)	-	−3.4	0.66
Summa 2009 [76]	Italy	RC	PVP	356	-	-	-	-	-	-	-	-	-	-	-
Tao 2024 [77]	China	RC	PKP (RAUPK)	151	19.3	5.6	73.5	7.6	45	82.12	23 (39.7)	36 (60.3)	-	−3.4	0.69
			PKP (FAUPK)		19.3	5.6	73.1	6.7	30		21 (36.8)	36 (36.2)	-	−3.4	0.52
			PKP (FABPK)		21.1	4.4	72.9	6.8	49		11 (30.6)	25 (69.4)	-	−3.2	0.52
Wang 2023 [79]	China	RC	PKP	313	-	-	61.9	8.7	91	49.7	84 (45.90)	99 (54.10)	-	-	-
			PVP		-	-	77.88	9.42	62	47.7	52 (40.00)	78 (60.00)	-	-	-
Wang 2024 [78]	China	RC	PKP	198	-	-	73.5	5.8	158	79.79	-	-	-	−2.6	0.73
Hey 2015 [44]	Singapore.	CC	PKP/PVP	93	25.1	21	76.8	13	76	81.7	69 (74.2)	97 (58.4)	-	-	-
Xinyu 2023 [81]	China	RC	PVP	300	-	-	72.2	5.7	232	77.3	-	-	-	−3.3	0.3
Yang 2020 [82]	Taiwan	RC	PVP	154	-	-	78.8	7.3	40	25.97	-	-	-	−2.5	0.99
Yao 2023 [83]	China	RC	PVP	113	-	-	70.7	7.5	24	70.79	1 (2.7)	33 (88.19)	-	-	-
			PVP		-	-	73.8	8.3	56		5 (6.25)	73 (87.95)	-	-	-
Yi 2014 [84]	China	RCT	PVP/PKP	290	94.4	11	61.3	6.8	181	62.41	145 (50.3)	217 (74.8)	-	-	-
Yin 2024 [85]	China	RC	PKP	101	-	-	67.3	7.3	51	50.5	-	-	-	-	-
Yu 2016 [86]	China	RC	PKP	104	28	3.3	74.3	9.34	70.7	68	-	-	-	−4.5	0.97
Yuntao 2025 [87]	China	RC	PVP	170	-	-	73.1	9.1	126	74.1	59 (34.7)	111 (65.3)	-	−3.3	0.91
Zhang 2011 [88]	China	RC	PKP	50	18	2.7	71.9	8.2	-	-	-	-	-	-	-
Zhang 2017 [91]	China	RC	PVP	66	-	-	71.3	7.2	44	66.7	38 (57.6)	28 (42.4)	-	-	-
Zhang 2019 [89]	China	RC	PVP	276	-	-	66.4	4.93	175	63.4	154 (55.79)	122 (44.2)	-	−3.1	0.41
Zhang 2021 [90]	China	RC	vertebral augmentation	23	21.5	-	74.4	11.5	14	60.9	-	-	-	−2.71	1.2
He 2021 [43]	China	RC	PKP	190	52.6	20	72.04	8.98	153	80.5	-	-	-	2.6	1.2
Zhong 2019 [92]	China	RC	PVP	104	-	-	72.9	8.1	89	85.6	7 (67.3)	12 (11.5)	98 (94.2)	-	-
Li 2020 [58]	China	RC	PVP	38	-	-	66.5	6.2	30	78.94	19 (50)	19 (50)	-	3.2	0.51
Zhuo 2022 [94]	China	RC	PVP	160	18.02	4.02	68.9	8.2	108	67.5	52 (32.5)	95 (59.4)	-	−4.1	2.5
Zhuo 2024 [95]	China	RCT	PVP	94	-	-	73.3	3.5	38	80.85	6 (12.77)	-	32 (68.1)	-	-
			PKP		-	-	71.86	3.77	39	82.97	5 (10.64)	-	35 (74.5)	-	-
Lin 2024 [60]	China	RC	PVP	111	-	-	-	-	-	-	-	-	-	-	-
Hillmeier 2004 [45]	China	RC	PKP	102	-	-	71	7.6	-	-	-	-	-	-	-
Pang 2019 [69]	China	RC	PKP	120	-	-	63.57	6.4	76	61.7	-	-	-	−3.2	1.2
Huang 2018 [48]	China	RC	PKP	187	-	-	-	-	-	-	10 (24.4)	-	22 (53.7)	-	-
Wu 2024 [80]	China	RC	PKP	115	6.74	-	-	-	-	-	-	-	-	-	-
Zhong-cheng 2022 [93]	China	RC	PKP	289	13.5	0.9	69.7	7.8	199	68.9	-	-	-	−2.8	1.3

YOP: year of publication; USA: United States of America; UK: United Kingdom; PKP: percutaneous kyphoplasty; PVP: percutaneous vertebroplasty; n: number of patients; SD: standard deviation; mo: month; FU: follow-up; RC: retrospective cohort; PC: prospective cohort; RCT: randomized controlled trial; CC: case–control; BMD: bone mineral density.

**Table 2:** A summary of the meta-analytic estimates (pooled rate) of re-fracture and mortality following surgical management of OVF.

	Subgroup	Re-Fracture Rate				Mortality Rate			
		Studies	Rate (95% CI)	I^2^ (%)	*p*-Value *	Studies	Rate (95% CI)	I^2^ (%)	*p*-Value *
Pooled	-	72	18 (15–21%)	99.33	-	23	15 (9–22%)	99.98	-
Country	Australia	3	15 (3–28%)	90.06	<0.001	4	8 (3–13%)	90.67	<0.001
	Bangladesh	1	31 (13–49%)	-		-	-	-	
	China	39	13 (10–15%)	93.51		3	20 (6–34%)	94.35	
	Germany	4	14 (9–18%)	73.44		3	1 (0–2%)	46.65	
	Italy	2	22 (5–40%)	98.54		1	1 (0–3%)	-	
	Japan	2	21 (12–31%)	62.2		1	5 (0–10%)	-	
	Singapore	1	17 (10–25%)	-		-	-	-	
	South Korea	2	20 (10–29%)	94.41		1	6 (6–6%)	-	
	Spain	1	10 (8–13%)	-		-	-	-	
	Taiwan	7	30 (12–48%)	98.8		1	40 (34–45%)	-	
	Turkey	1	50 (12–88%)	-		1	33 (24–42%)	-	
	UK	1	3 (0–6%)	-		2	10 (0–22%)	90.55	
	USA	7	21 (10–32%)	98.5		4	25 (7–42%)	99.96	
Study Design	Case–control	1	17 (10–25%)	-	0.95	1	4 (4–4%)	-	<0.001
	Prospective cohort	11	16 (10–22%)	94.92		5	6 (1–12%)	97.39	
	RCT	9	17 (9–25%)	95.34		3	5 (3–8%)	41.43	
	Retrospective cohort	52	18 (15–21%)	99.55		14	22 (12–31%)	99.99	
Surgery Type	PKP	33	16 (13–20%)	94.9	0.37	7	14 (4–24%)	99.39	0.51
	PVP	41	19 (14–24%)	99.13		12	19 (8–31%)	99.84	
Surgical Approach	Bilateral	17	17 (10–24%)	96.96	0.56	5	1 (0–1%)	0.00	<0.001
	Planned Puncture	3	18 (9–26%)	69.83		-	-	-	
	Transpedicular	3	13 (11–15%)	24.62		1	25 (22–28%)	-	
	Unilateral	6	15 (3–27%)	98.23		2	38 (0–87%)	95.45	

* *p*-value for between subgroup comparison; CI: confidence interval; PKP: percutaneous kyphoplasty; PVP: percutaneous vertebroplasty; UK: United Kingdom; USA: United States of America; I^2^: a measure of statistical heterogeneity, where significant heterogeneity is defined by I^2^ > 50%; OVF: osteoporotic vertebral fracture.

**Table 3:** Multivariate adjusted meta-regression analysis of the determinants of re-fracture rate following surgical management of OVF.

	Coefficient	SE	z	*p*-Value	Low CI	High CI
Mean BMD (per point increase)	−0.032	0.056	−0.560	0.575	−0.142	0.079
Prior Fracture History (per % increase)	−0.003	0.002	−1.600	0.110	−0.006	0.001
Cement volume (per unit increase)	0.003	0.030	0.100	0.917	−0.056	0.062
Cement Leak (per % increase)	0.002	0.001	2.770	0.006	0.001	0.003

In the unadjusted model, age, female, interval from injury to surgery, diabetes, operative time, anterior and posterior vertebral heights, kyphosis angle, and preoperative pain (VAS) and ODI scores were added, all of which were deemed insignificant (*p* < 0.01). VAS: visual analogue scale; BMD: bone min density; SE: standard error; CI: confidence interval; ODI: Oswestry Disability Index; OVF: osteoporotic vertebral fracture.

**Table 4:** Univariate meta-regression analysis of the determinants of mortality rate following surgical management of OVF.

	Coefficient	SE	z	*p*-Value	Low CI	High CI
Mean age (per year increase)	0.000	0.007	−0.040	0.971	−0.013	0.013
Female (per % increase)	−0.002	0.003	−0.730	0.466	−0.007	0.003
Mean BMD (per point increase)	0.015	0.011	1.390	0.165	−0.006	0.036
Prior Fracture History (per % increase)	−0.006	0.002	−3.590	0.0001	−0.010	−0.003
Interval from Injury to Surgery (per month increase)	−0.011	0.007	−1.610	0.107	−0.023	0.002
DM (per % increase)	0.019	0.006	3.350	0.001	0.008	0.031
Cement volume (per unit increase)	0.000	0.024	−0.010	0.993	−0.048	0.048
Cement Leak (per % increase)	−0.001	0.002	−0.800	0.426	−0.005	0.002
Operative time (per minute increase)	0.001	0.001	1.230	0.220	−0.001	0.002
Preop VAS Score (per unit increase)	0.000	0.001	0.140	0.892	−0.002	0.002
Preop ODI score (per unit increase)	0.001	0.001	0.820	0.414	−0.001	0.002

Variables like anterior and posterior vertebral heights and kyphosis angle were excluded from the analysis due to small sample size (<10 studies per covariate). A multivariate model adjusting for variables with *p*-value < 0.10 was not done due to small sample (underpowered analysis; <10 studies per each covariate). VAS: visual analogue scale; BMD: bone min density; DM: diabetes mellitus; SE: standard error; CI: confidence interval; ODI: Oswestry Disability Index; OVF: osteoporotic vertebral fracture.

## Data Availability

Data are within the manuscript and its Supplementary Materials.

## Supplementary Materials

The following supporting information can be downloaded at: https://www.mdpi.com/article/10.3390/jcm14228230/s1, Figure S1: Forest plot showing the pooled refracture rate in patients with surgically treated OVF [14,15,16,18,19,21,22,23,24,25,26,27,29,30,31,32,33,34,35,36,37,39,40,41,42,43,44,45,46,47,48,49,50,51,52,53,54,58,59,60,61,62,63,64,65,66,67,68,70,71,72,73,74,75,76,77,78,79,81,82,83,85,86,87,88,89,91,92,93,94,95]; Figure S2: Leave-one-out sensitivity analysis of the pooled refracture rate in patients with surgically treated OVF [14,15,16,18,19,21,22,23,24,25,26,27,29,30,31,32,33,34,35,36,37,39,40,41,42,43,44,45,46,47,48,49,50,51,52,53,54,58,59,60,61,62,63,64,65,66,67,68,70,71,72,73,74,75,76,77,78,79,81,82,83,85,86,87,88,89,91,92,93,94,95]; Figure S3: Galbraith plot of the pooled refracture rate in patients with surgically treated OVF; Figure S4: Funnel plot showing the risk of bias in the pooled refracture rate in patients with surgically treated OVF; Figure S5: Reconstructed Kaplan–Meier Curve showing the time-to-refracture in patients with OVF; Figure S6: Forest plot showing the pooled mortality rate in patients with surgically treated OVF [17,18,20,22,26,28,32,37,38,43,51,52,53,54,55,56,57,64,65,66,68,71,85]; Figure S7: Leave-one-out sensitivity analysis of the pooled mortality rate in patients with surgically treated OVF [17,18,20,22,26,28,32,37,38,43,51,52,53,54,55,56,57,64,65,66,68,71,85]; Figure S8: Galbraith plot of the pooled mortality rate in patients with surgically treated OVF; Figure S9: Funnel plot showing the risk of bias in the pooled mortality rate in patients with surgically treated OVF; Figure S10: Reconstructed Kaplan–Meier Curve showing the survival time (time-to-death) in patients with OVF; Table S1: The search query employed in the database search; Table S2: The summary of the methodological quality of included non-randomized studies using the National Institute of Health (NIH) tool; Table S3: An exploratory analysis of the pooled rate of adjacent and non-adjacent re-fractures following the surgical management of OVF; Supplementary checklist: A filled-out PRISMA-compliant checklist for systematic reviews.

## Abbreviations

The following abbreviations are used in this manuscript:

OVF	Osteoporotic Vertebral Fracture
PVP	Percutaneous Vertebroplasty
PKP	Percutaneous Kyphoplasty
BMD	Bone Mineral Density
HR	Hazard Ratio
CI	Confidence Interval
PRISMA	Preferred Reporting Items for Systematic Reviews and Meta-Analyses
RCT	Randomized Controlled Trial
IPD	Individual Patient Data
RoB 2	Risk of Bias 2.0
NIH	National Institutes of Health
ODI	Oswestry Disability Index
VAS	Visual Analog Scale
SD	Standard Deviation
