# Supplementary material for: Refracture and Mortality Following Surgical Management of Osteoporotic Vertebral Fractures: A Systematic Review and Meta-Analysis with Patient-Level Survival Modeling
Source: J Clin Med. 2025 Nov 20;14(22):8230. doi: 10.3390/jcm14228230 (PMC12653041; doi:10.3390/jcm14228230)
Supplement: Supplementary file 1 [file jcm-14-08230-s001.zip › Figure S2.pdf]

Omitted study	Proportion with 95% CI	p-value
Ahsan 2021	0.18 [0.14, 0.21]	0.000
Ali 2009	0.18 [0.15, 0.21]	0.000
Bae 2017	0.18 [0.15, 0.21]	0.000
Beall 2019	0.17 [0.14, 0.20]	0.000
Becker 2007	0.18 [0.15, 0.21]	0.000
Benedict 2025	0.18 [0.15, 0.21]	0.000
Bergmann 2012	0.18 [0.15, 0.21]	0.000
Bu 2022	0.17 [0.14, 0.21]	0.000
Chang 2020	0.18 [0.15, 0.21]	0.000
Chang 2020	0.18 [0.15, 0.21]	0.000
Chen 2010	0.18 [0.15, 0.21]	0.000
Chen 2015	0.18 [0.15, 0.21]	0.000
Chen 2017b	0.17 [0.14, 0.20]	0.000
Chen 2023	0.18 [0.15, 0.21]	0.000
Chi 2020	0.18 [0.15, 0.21]	0.000
Chi 2020	0.18 [0.15, 0.21]	0.000
Chien 2021	0.18 [0.14, 0.21]	0.000
Clark 2016	0.18 [0.15, 0.21]	0.000
Dai 2021	0.18 [0.15, 0.21]	0.000
Dai 2021	0.18 [0.15, 0.21]	0.000
Dai 2024	0.18 [0.15, 0.21]	0.000
Goldman-Daleo 2023	0.18 [0.15, 0.21]	0.000
Deng 2018	0.18 [0.15, 0.21]	0.000
Diamond 2006	0.18 [0.15, 0.21]	0.000
Gan 2013	0.18 [0.15, 0.21]	0.000
Gan 2013	0.18 [0.15, 0.21]	0.000
Gonzalez 2023	0.18 [0.15, 0.21]	0.000
Guo 2021	0.18 [0.15, 0.21]	0.000
Hu 2019	0.18 [0.15, 0.21]	0.000
Huang 2021	0.18 [0.15, 0.21]	0.000
Huntoon 2008	0.17 [0.14, 0.20]	0.000
Jue 2021	0.18 [0.15, 0.21]	0.000
Kang 2022	0.18 [0.15, 0.21]	0.000
Kara 2023	0.17 [0.14, 0.20]	0.000
Kara 2023	0.18 [0.14, 0.21]	0.000
Kato 2020	0.18 [0.15, 0.21]	0.000
Kim 2014	0.18 [0.15, 0.21]	0.000
Li 2024	0.18 [0.15, 0.21]	0.000
Lin 2016	0.18 [0.15, 0.21]	0.000
Lin 2016	0.18 [0.15, 0.21]	0.000
Röllinghoff 2009	0.18 [0.15, 0.21]	0.000
Röllinghoff 2009	0.18 [0.15, 0.21]	0.000
Ma 2021	0.18 [0.15, 0.21]	0.000
Matsumoto 2024	0.18 [0.15, 0.21]	0.000
Mazzantini 2020	0.17 [0.14, 0.20]	0.000
DePalma 2011	0.18 [0.15, 0.21]	0.000
Moulin 2020	0.18 [0.15, 0.21]	0.000
Mukherjee 2015	0.18 [0.15, 0.21]	0.000
Ning 2021	0.18 [0.15, 0.21]	0.000
Noriega 2019	0.17 [0.14, 0.21]	0.000
Noriega 2019	0.17 [0.14, 0.21]	0.000
Pflugmacher 2006	0.18 [0.15, 0.21]	0.000
Pitton 2018	0.18 [0.15, 0.21]	0.000
Pitton 2018	0.18 [0.15, 0.21]	0.000
Qi 2024	0.18 [0.15, 0.21]	0.000
Qian 2022	0.18 [0.15, 0.21]	0.000
Song 2023	0.18 [0.15, 0.21]	0.000
Summa 2009	0.18 [0.15, 0.21]	0.000
Summa 2009	0.18 [0.14, 0.21]	0.000
Summa 2009	0.18 [0.15, 0.21]	0.000
Tao 2024	0.18 [0.15, 0.21]	0.000
Tao 2024	0.18 [0.15, 0.21]	0.000
Tao 2024	0.18 [0.15, 0.21]	0.000
Wang 2023	0.18 [0.15, 0.21]	0.000
Wang 2024	0.18 [0.15, 0.21]	0.000
Hey 2015	0.18 [0.15, 0.21]	0.000
Xinyu 2023	0.18 [0.14, 0.21]	0.000
Yang 2020	0.17 [0.14, 0.19]	0.000
Yao 2023	0.18 [0.15, 0.21]	0.000
Yao 2023	0.18 [0.15, 0.21]	0.000
Yin 2024	0.18 [0.15, 0.21]	0.000
Yu 2016	0.18 [0.15, 0.21]	0.000
Yuntao 2025	0.18 [0.15, 0.21]	0.000
Zhang 2011	0.18 [0.15, 0.21]	0.000
Zhang 2017	0.18 [0.15, 0.21]	0.000
Zhang 2019	0.18 [0.15, 0.21]	0.000
He 2021	0.17 [0.14, 0.20]	0.000
Zhong 2019	0.18 [0.15, 0.21]	0.000
Li 2020	0.18 [0.14, 0.21]	0.000
Zhuo 2022	0.18 [0.15, 0.21]	0.000
Zhuo 2024	0.18 [0.15, 0.21]	0.000
Zhuo 2024	0.18 [0.15, 0.21]	0.000
Lin 2024	0.18 [0.15, 0.21]	0.000
Hillmeier 2004	0.18 [0.15, 0.21]	0.000
Huang 2018	0.18 [0.15, 0.21]	0.000
Zhong-cheng 2022	0.18 [0.15, 0.21]	0.000

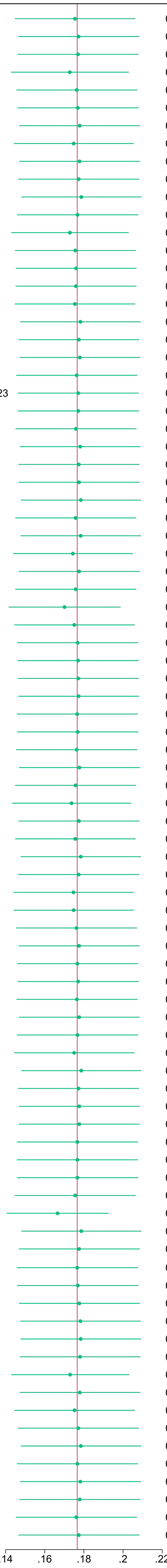

Random-effects REML model
