# Supplementary material for: Refracture and Mortality Following Surgical Management of Osteoporotic Vertebral Fractures: A Systematic Review and Meta-Analysis with Patient-Level Survival Modeling
Source: J Clin Med. 2025 Nov 20;14(22):8230. doi: 10.3390/jcm14228230 (PMC12653041; doi:10.3390/jcm14228230)
Supplement: Supplementary file 1 [file jcm-14-08230-s001.zip › Supplementary tables.pdf]

Table S1. The search query employed in the database search

No.	Search query	Results
**PubMed**		
**#1**	Mortality[tiab] OR death[tiab] OR died[tiab] OR refracture[tiab] OR "re-fracture"[tiab] OR "additional fracture"[tiab]	2072097
**#2**	Osteoporotic[tiab]	25656
**#3**	Vertebral[tiab] OR spinal[tiab]	402473
**#4**	Fracture*[tiab]	343110
**#5**	"risk factor*"[tiab] OR "relative risk"[tiab] OR regression[tiab] OR determinant*[tiab] OR predictor*[tiab] OR factor*[tiab] OR multivariate[tiab] OR multivariable[tiab] OR univariate[tiab] OR univariable[tiab] OR binomial[tiab] OR "odds ratio"[tiab] OR hazard*[tiab] OR Prevalen*[tiab] OR proportion[tiab] OR frequen*[tiab] OR incidence[tiab] OR rate[tiab] OR percentage[tiab] OR occurrence[tiab] OR occurred[tiab]	11722707
**#6**	Surgical[tiab] OR surgery[tiab] OR operation[tiab] OR operative[tiab] OR vertebroplasty[tiab] OR kyphoplasty[tiab] OR augmentation[tiab] OR "spinal fusion"[tiab] OR fixation[tiab] OR instrumentation[tiab]	3005343
**#7**	#1 AND #2 AND #3 AND #4 AND #5 AND #6	281
**Scopus**		
**#1**	TITLE-ABS-KEY (Mortality) OR TITLE-ABS-KEY (death) OR TITLE-ABS-KEY (died) OR TITLE-ABS-KEY (refracture) OR TITLE-ABS-KEY ("re-fracture") OR TITLE-ABS-KEY ("additional fracture")	3545402
**#2**	TITLE-ABS-KEY (Osteoporotic)	34557
**#3**	TITLE-ABS-KEY (Vertebral) OR TITLE-ABS-KEY (spinal)	660380
**#4**	TITLE-ABS-KEY (Fracture*)	1080387
**#5**	TITLE-ABS-KEY ("risk factor*") OR TITLE-ABS-KEY ("relative risk") OR TITLE-ABS-KEY (regression) OR TITLE-ABS-KEY (determinant*) OR TITLE-ABS-KEY (predictor*) OR TITLE-ABS-KEY (factor*) OR TITLE-ABS-KEY (multivariate) OR TITLE-ABS-KEY (multivariable) OR TITLE-ABS-KEY (univariate) OR TITLE-ABS-KEY (univariable) OR TITLE-ABS-KEY (binomial) OR TITLE-ABS-KEY ("odds ratio") OR TITLE-ABS-KEY (hazard*) OR TITLE-ABS-KEY (Prevalen*) OR TITLE-ABS-KEY (proportion) OR TITLE-ABS-KEY (frequen*) OR TITLE-ABS-KEY (incidence) OR TITLE-ABS-KEY (rate) OR TITLE-ABS-KEY (percentage) OR TITLE-ABS-KEY (occurrence) OR TITLE-ABS-KEY (occurred)	30176756
**#6**	TITLE-ABS-KEY (Surgical) OR TITLE-ABS-KEY (surgery) OR TITLE-ABS-KEY (operation) OR TITLE-ABS-KEY (operative) OR TITLE-ABS-KEY (vertebroplasty) OR TITLE-ABS-KEY (kyphoplasty) OR TITLE-ABS-KEY (augmentation) OR TITLE-ABS-KEY ("spinal fusion") OR TITLE-ABS-KEY (fixation) OR TITLE-ABS-KEY (instrumentation)	7626170
**#7**	#1 AND #2 AND #3 AND #4 AND #5 AND #6	469
**Web of Science**		
**#1**	AB=Mortality OR AB=death OR AB=died OR AB=refracture OR AB="re-fracture" OR AB="additional fracture"	2279838
**#2**	AB=Osteoporotic	22272
**#3**	AB=Vertebral OR AB=spinal	321536

**#4**	AB=Fracture*	522438
**#5**	AB="risk factor*" OR AB="relative risk" OR AB=regression OR AB=determinant* OR AB=predictor* OR AB=factor* OR AB=multivariate OR AB=multivariable OR AB=univariate OR AB=univariable OR AB=binomial OR AB="odds ratio" OR AB=hazard* OR AB=Prevalen* OR AB=proportion OR AB=frequen* OR AB=incidence OR AB=rate OR AB=percentage OR AB=occurrence OR AB=occurred	21522681
**#6**	AB=Surgical OR AB=surgery OR AB=operation OR AB=operative OR AB=vertebroplasty OR AB=kyphoplasty OR AB=augmentation OR AB="spinal fusion" OR AB=fixation OR AB=instrumentation	3707686
**#7**	#1 AND #2 AND #3 AND #4 AND #5 AND #6	232

Table S2. The summary of the methodological quality of included non-randomized studies using the National Institute of Health (NIH) tool

Study	Q1	Q2	Q3	Q4	Q5	Q6	Q7	Q8	Q9	Q10	Q11	Q12	Q13	Q14	Total score	Overall Quality
**Ahsan 2021 [14]**	1	2	2	1	0	2	2	1	2	1	2	0	2	0	18	Fair
**Ali 2009 [15]**	1	2	2	1	0	1	0	1	2	1	2	0	2	0	15	Fair
**Bae 2017 [16]**	2	2	1	1	0	1	2	1	2	1	2	0	1	0	16	Fair
**Banat 2022 [17]**	1	2	2	2	0	1	2	1	2	1	2	0	2	0	18	Fair
**Becker 2011 [20]**	1	2	2	2	0	1	0	1	2	1	2	0	2	0	16	Fair
**Benedict 2025 [21]**	1	2	1	2	0	1	2	1	2	1	2	0	2	0	17	Fair
**Bergmann 2012 [22]**	2	2	2	1	0	1	2	1	2	1	2	0	2	0	18	Fair
**Bu 2022 [23]**	1	2	2	1	0	1	2	1	2	1	2	0	2	0	17	Fair
**Chen 2015 [25]**	1	2	2	0	0	2	2	2	2	1	2	0	2	0	18	Fair
**Chen 2017a [28]**	2	2	2	2	0	1	2	2	2	1	2	0	2	0	20	Fair
**Chen 2017b [29]**	2	2	2	1	0	1	2	1	2	1	2	0	2	0	18	Fair
**Chen 2023 [27]**	2	2	2	2	0	1	2	1	2	1	2	0	2	0	19	Fair
**Chi 2020 [30]**	2	2	2	2	0	1	2	1	2	1	2	0	2	0	19	Fair
**Chien 2021 [31]**	1	2	2	2	0	1	2	1	2	1	2	0	2	0	18	Fair
**Goldman-Daelo 2023 [40]**	1	2	1	1	0	1	2	1	2	1	2	0	1	0	15	Fair
**Dai 2024 [34]**	2	2	2	2	0	1	2	1	2	1	2	0	2	0	19	Fair
**Diamond 2006 [37]**	2	2	2	0	0	1	2	1	2	2	2	0	2	0	18	Fair
**Edidin 2015 [38]**	2	2	2	1	0	1	2	2	2	2	2	1	2	0	21	Good
**Gan 2013 [39]**	1	2	2	2	0	1	2	1	2	1	2	0	2	0	18	Fair
**Gonzalez 2023 [42]**	2	2	2	1	0	1	2	1	2	1	2	0	2	0	18	Fair
**Guo 2021 [41]**	2	2	2	0	0	1	2	1	2	2	2	0	2	0	18	Fair
**Hillmeier 2004 [45]**	2	2	2	0	0	1	2	1	2	2	2	0	2	0	18	Fair
**Hu 2019 [46]**	2	2	2	1	0	1	2	1	2	1	2	0	2	0	18	Fair
**Huang 2021 [47]**	2	2	2	2	0	1	2	2	2	2	2	1	2	0	22	Good
**Huntoon 2008 [49]**	2	2	0	1	0	1	2	1	2	1	2	1	0	0	15	Fair
**Jue 2021 [50]**	1	2	1	1	0	1	2	1	2	1	2	0	1	0	15	Fair
**Kang 2022 [51]**	2	2	2	2	0	1	2	2	2	2	2	2	1	0	22	Good

**Kara 2023 [52]**	1	2	2	1	0	1	2	1	2	1	2	0	2	0	17	Fair
**Kato 2020 [53]**	2	2	2	1	0	1	2	1	2	2	2	0	2	2	21	Good
**Kim 2014 [54]**	1	2	2	0	0	1	2	2	2	1	2	0	2	0	17	Fair
**Kim 2022 [55]**	2	1	2	1	0	1	2	1	2	2	1	0	2	2	19	Fair
**Klezel 2012 [56]**	2	2	2	1	0	1	2	1	2	2	2	0	2	0	19	Fair
**Leslie 2013 [57]**	2	2	2	0	0	1	2	1	2	2	2	0	2	0	18	Fair
**Li 2024 [59]**	2	2	2	1	0	2	2	2	2	2	2	0	2	0	21	Fair
**Lin 2016 [61]**	2	2	2	1	0	1	2	1	2	1	2	1	2	2	21	Good
**Lin 2024 [60]**	1	2	2	0	0	1	2	2	2	1	2	0	2	0	17	Fair
**Ma 2021 [62]**	2	2	2	1	0	2	2	1	2	2	2	0	2	0	20	Fair
**Matsumoto 2024 [63]**	2	2	2	1	0	1	2	1	2	1	2	1	2	2	21	Good
**Mazzantini 2020 [64]**	2	2	2	1	0	2	2	2	2	1	2	0	2	2	22	Good
**DePalma 2011 [36]**	2	2	2	1	0	1	2	1	2	2	2	0	2	0	19	Fair
**Moulin 2020 [65]**	1	2	2	0	0	1	2	2	2	1	2	0	2	0	17	Fair
**Mukherjee 2015 [66]**	2	2	2	2	0	2	2	2	2	2	2	0	2	0	22	Good
**Ning 2021 [67]**	2	2	2	1	0	1	2	1	2	2	2	0	2	0	19	Fair
**Pflugmacher 2006 [70]**	2	2	1	2	0	2	2	2	2	2	2	0	2	0	21	Good
**Pitton 2018 [71]**	2	2	2	1	0	1	2	2	1	2	2	0	2	0	19	Fair
**Qi 2024 [72]**	2	2	2	1	0	1	2	1	2	1	2	1	2	2	21	Good
**Qian 2022 [73]**	2	2	2	0	0	1	2	1	2	1	2	0	2	0	17	Fair
**Röllinghoff 2009 [74]**	2	2	2	0	0	1	2	1	2	2	2	0	2	0	18	Fair
**Song 2023 [75]**	2	2	2	1	0	1	2	1	2	1	2	1	2	0	19	Fair
**Summa 2009 [76]**	1	2	2	0	0	1	2	2	2	1	2	0	2	0	17	Fair
**Tao 2024 [77]**	2	2	2	2	0	1	2	1	2	1	2	1	2	0	20	Fair
**Huang 2018 [48]**	2	2	1	1	0	1	2	1	2	1	2	0	1	0	16	Fair
**Hey 2015 [44]**	2	2	2	1	0	1	2	1	2	1	2	0	2	0	18	Fair
**Wang 2023 [79]**	2	2	2	2	0	1	0	1	2	2	2	1	2	0	19	Fair
**Wang 2024 [78]**	2	2	2	0	0	1	2	1	2	2	2	0	2	0	18	Fair
**Xinyu 2023 [81]**	2	2	1	2	0	1	2	2	2	2	2	1	2	0	21	Good
**Yang 2020 [82]**	2	2	2	2	0	1	0	1	2	2	2	1	2	0	19	Fair

**Yao 2023 [83]**	2	2	2	2	0	1	2	2	2	2	2	0	2	0	21	Good
**Yin 2024 [85]**	2	2	2	2	0	1	0	1	2	2	2	1	2	0	19	Fair
**Wu 2024 [80]**	1	2	2	1	0	2	2	1	2	1	2	0	2	0	18	Fair
**Yu 2016 [86]**	2	2	2	2	0	1	0	1	2	2	2	1	2	0	19	Fair
**Yuntao 2025 [87]**	2	2	2	2	0	1	2	2	2	2	2	1	2	0	22	Good
**Zhang 2011 [88]**	1	2	2	0	0	1	2	2	2	1	2	0	2	0	17	Fair
**Zhang 2017 [91]**	2	2	2	0	0	1	2	2	2	1	2	0	2	0	18	Fair
**Zhang 2019 [89]**	2	2	2	1	0	1	2	2	2	2	2	1	2	0	21	Good
**Zhang 2021 [90]**	2	2	2	1	0	1	2	2	2	2	2	1	2	0	21	Good
**He 2021 [43]**	1	2	2	0	0	1	2	2	2	1	2	0	2	0	17	Fair
**Zhong 2019 [92]**	2	2	2	2	0	1	2	1	2	1	2	1	2	0	20	Fair
**Li 2020 [58]**	1	2	2	1	0	2	2	1	2	1	2	0	2	0	18	Fair
**Zhong-cheng 2022 [93]**	2	2	2	0	0	1	2	1	2	2	2	0	2	0	18	Fair
**Zhuo 2022 [94]**	2	2	2	2	0	1	2	1	2	2	2	1	2	0	21	Good
**Zhuo 2024 [95]**	2	2	2	2	0	1	2	1	2	2	2	1	2	0	21	Good

Q1. Was the research question or objective in this paper clearly stated? Q2. Was the study population clearly specified and defined? Q3. Was the participation rate of eligible persons at least 50%? Q4. Were all the subjects selected or recruited from the same or similar populations (including the same time period)? Were the inclusion and exclusion criteria for being in the study prespecified and applied uniformly to all participants? Q5. Was a sample size justification, power description, or variance and effect estimates provided? Q6. For the analyses in this paper, were the exposure(s) of interest measured prior to the outcome(s) being measured? Q7. Was the timeframe sufficient so that one could reasonably expect to see an association between exposure and outcome if it existed? Q8. For exposures that can vary in amount or level, did the study examine different levels of the exposure as related to the outcome (e.g., categories of exposure, or exposure measured as a continuous variable)? Q9. Were the exposure measures (independent variables) clearly defined, valid, reliable, and implemented consistently across all study participants? Q10. Were the exposure(s) assessed more than once over time? Q11. Were the outcome measures (dependent variables) clearly defined, valid, reliable, and implemented consistently across all study participants? Q12. Were the outcome assessors blinded to the exposure status of participants? Q13. Was the loss to follow-up after baseline 20% or less? Q14. Were key potential confounding variables measured and adjusted statistically for their impact on the relationship between exposure(s) and outcome(s)?

Table S3. An exploratory analysis of the pooled rate of adjacent and non-adjacent re-fractures following the surgical management of OVF

	Subgroup	Adjacent Fracture				Non-adjacent Fracture			
		Studies	Rate (95% CI)	I^2 (%)	P-value*	Studies	Rate (95% CI)	I^2 (%)	P-value*
**Pooled**	-	37	12.1 (9.2 - 14.9%)	98.06	-	21	8.8 (5.4 - 12.2%)	96.98	-
**Country**	Australia	2	12.2 (6.5 - 18%)	24.36	0.0001	1	3.3 (0 - 7.9%)	24.36	0.0001
	Bangladesh	1	23.1 (6.9 - 39.3%)	-		1	15.4 (1.5 - 29.3%)	-	
	China	12	9.9 (6.6 - 13.1%)	90.45		6	6.8 (5.4 - 13.9%)	78.49	
	Germany	3	15.3 (2.7 - 27.9%)	89.05		1	13.2 (7.4 - 19%)	-	
	Japan	1	7.5 (3 - 12%)	-		0	-	-	
	Singapore	1	7.5 (2.2 - 12.9%)	-		0	-	-	
	South Korea	1	9.9 (6 - 13.7%)	-		1	4.7 (2 - 7.4%)	-	
	Spain	1	4 (2.4 - 5.6%)	-		0	-	-	
	Taiwan	4	18.4 (1.6 - 35.2%)	98.46		2	3.4 (0.4 - 6.5%)	0	
	USA	5	13.7 (5.7 - 21.8%)	95.51		4	12.1 (0 - 27.2%)	99.68	
**Study Design**	Case-control	1	7.5 (2.2 - 12.9%)	-	0.249	0	-	-	0.249
	Prospective cohort	6	14.1 (8.1 - 20.2%)	80.81		3	11.4 (6.6 - 20.9%)	70.64	
	RCT	5	15.5 (8.1 - 22.9%)	87.73		3	9.2 (2.6 - 15.9%)	84.75	
	Retrospective cohort	20	10.6 (7 - 14.1%)	98.72		11	7.8 (2.9 - 12.8%)	98.37	
**Surgery Type**	PKP	18	13.2 (9.3 - 17.2%)	95.55	0.894	8	5.3 (3.1 - 7.6%)	81.62	0.243
	PVP	15	12.8 (7 - 18.5%)	97.87		10	9.3 (3.1 - 15.5%)	96.73	
**Surgical Approach**	Bilateral	8	14.6 (7.7 - 21.6%)	92.98	0.0001	3	9.6 (6.5 - 12.7%)	0.02	0.0001
	Planned Puncture	1	15.4 (8.5 - 22.3%)	-		0	-	-	
	Transpedicular	3	9.4 (4.3 - 14.4%)	88.73		2	5.3 (1.6 - 9.1%)	91.72	
	Unilateral	2	1.6 (0 - 4%)	75.89		1	2.1 (0.1 - 4.1%)	-	

*p-value for between subgroup comparison; CI: confidence interval; PKP: percutaneous kyphoplasty; PVP: percutaneous vertebroplasty; USA: United States of America; I^2: a measure of statistical heterogeneity, where significant heterogeneity is defined by $I^2 > 50\%$; OVF: osteoporotic vertebral fracture.
