# Supplementary figures and images for: Refracture and Mortality Following Surgical Management of Osteoporotic Vertebral Fractures: A Systematic Review and Meta-Analysis with Patient-Level Survival Modeling
Source: J Clin Med. 2025 Nov 20;14(22):8230. doi: 10.3390/jcm14228230 (PMC12653041; doi:10.3390/jcm14228230)

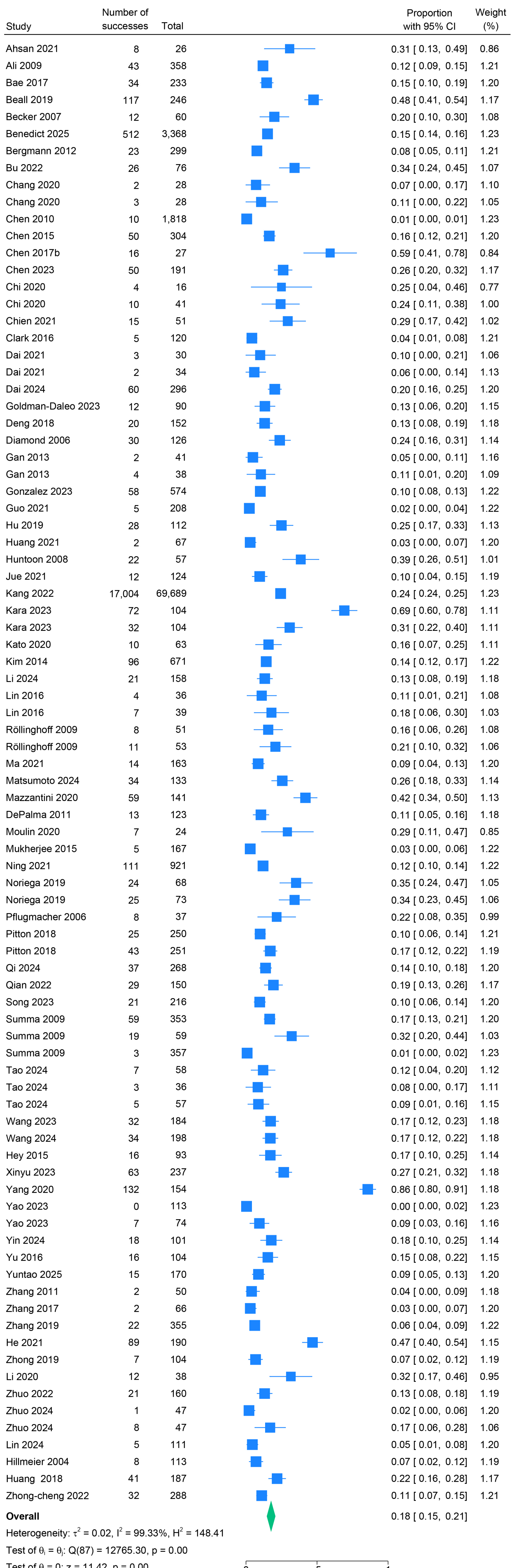

Random-effects REML model

Supplement: Supplementary file 1 [file jcm-14-08230-s001.zip › Figure S1.pdf]

# Kaplan–Meier survival estimate

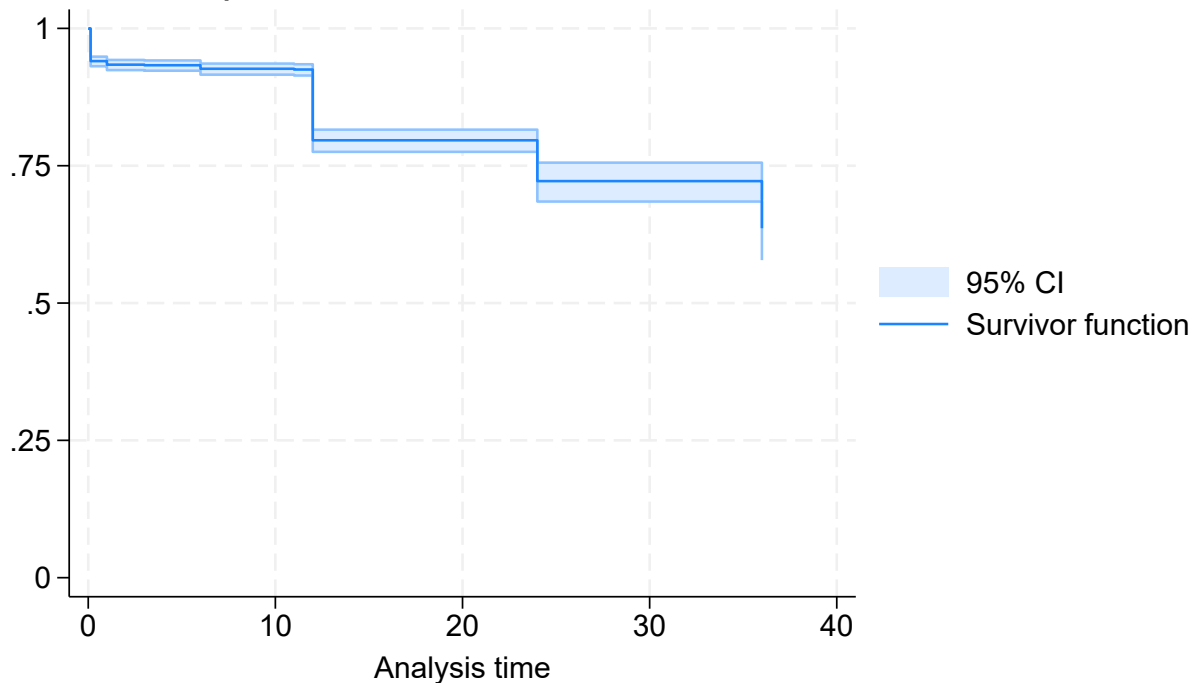

Number at risk

2888

1333

225

101

0

Supplement: Supplementary file 1 [file jcm-14-08230-s001.zip › Figure S10.pdf]

Galbraith plot

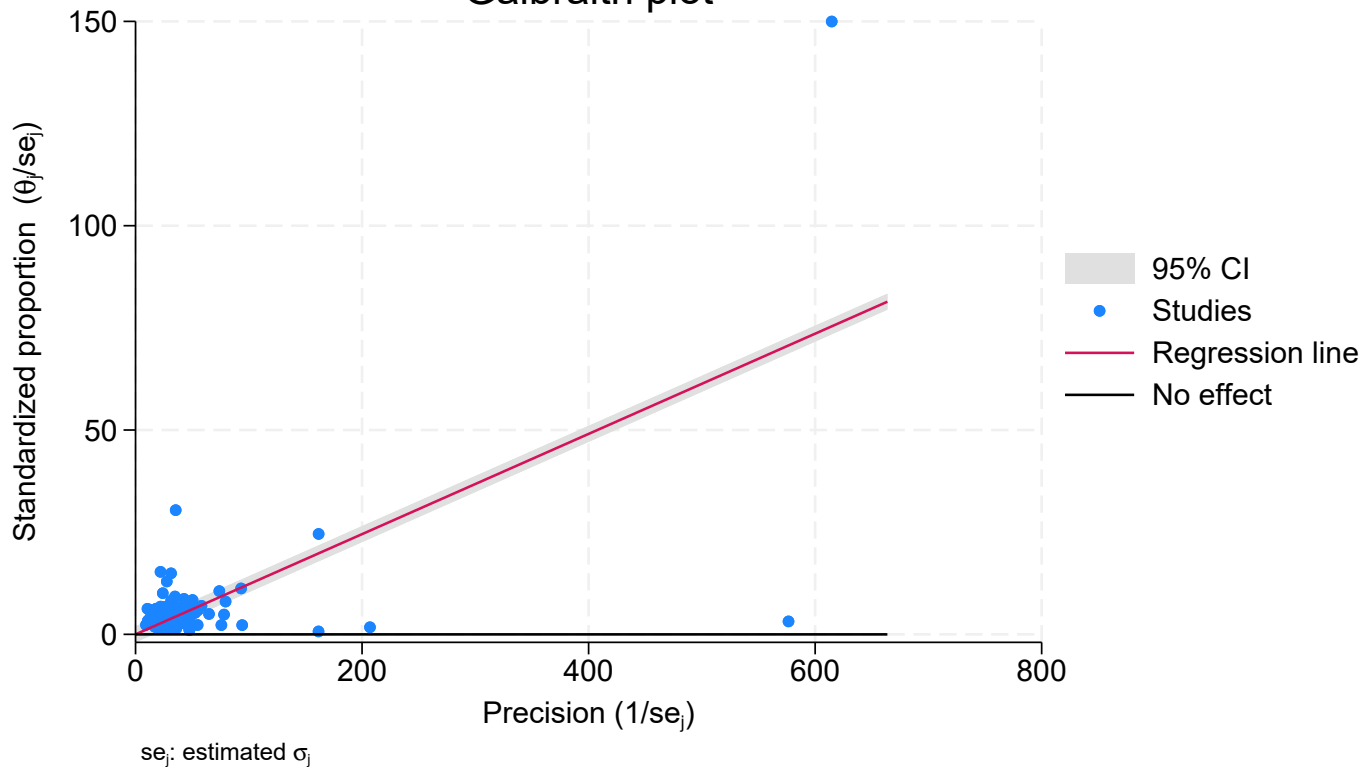

Supplement: Supplementary file 1 [file jcm-14-08230-s001.zip › Figure S3.pdf]

Funnel plot

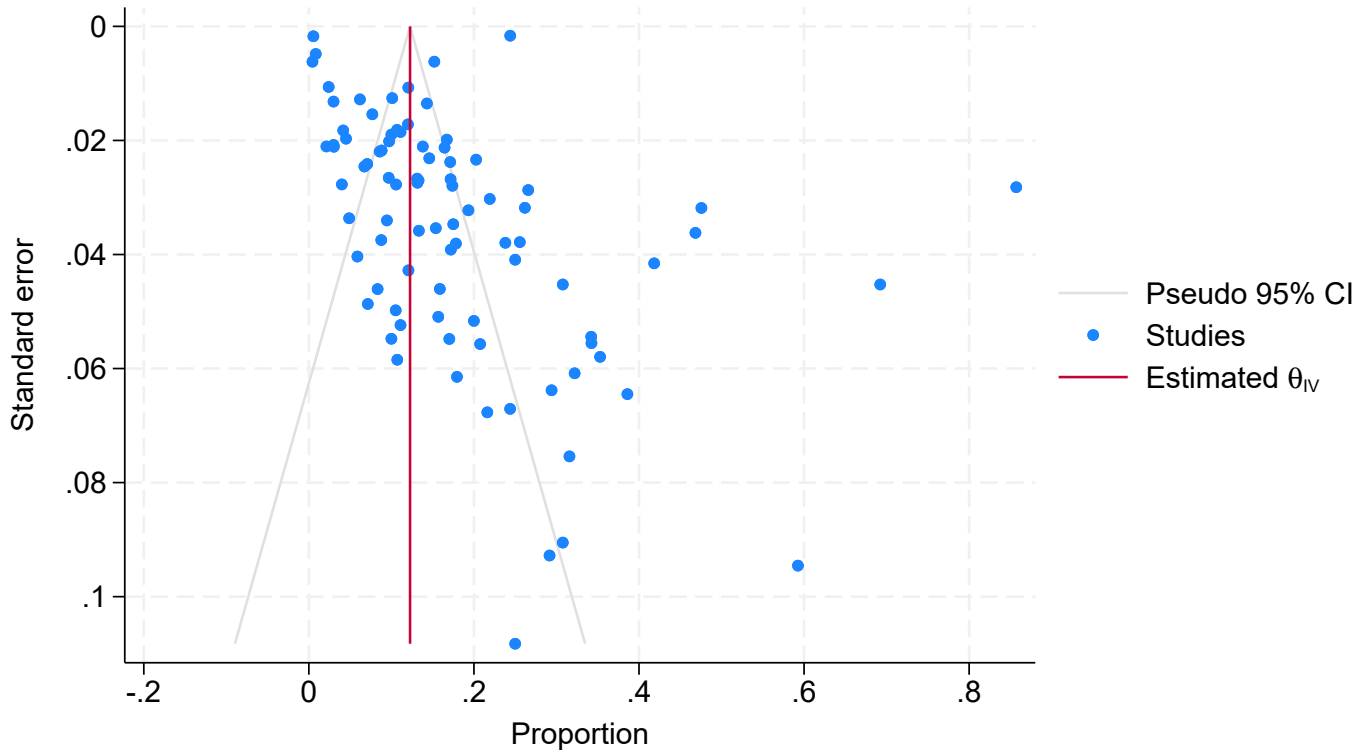

Supplement: Supplementary file 1 [file jcm-14-08230-s001.zip › Figure S4.pdf]

## Kaplan–Meier failure estimate

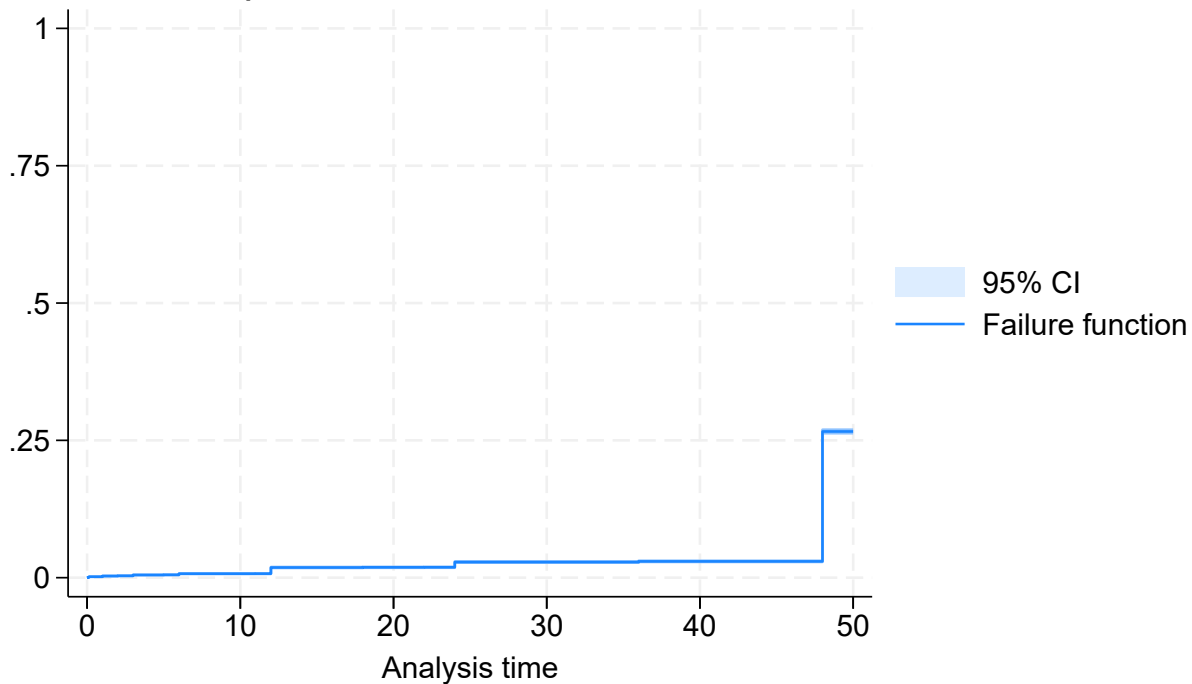

Number at risk

87257

83496

77504

70658

70130

251

Supplement: Supplementary file 1 [file jcm-14-08230-s001.zip › Figure S5.pdf]

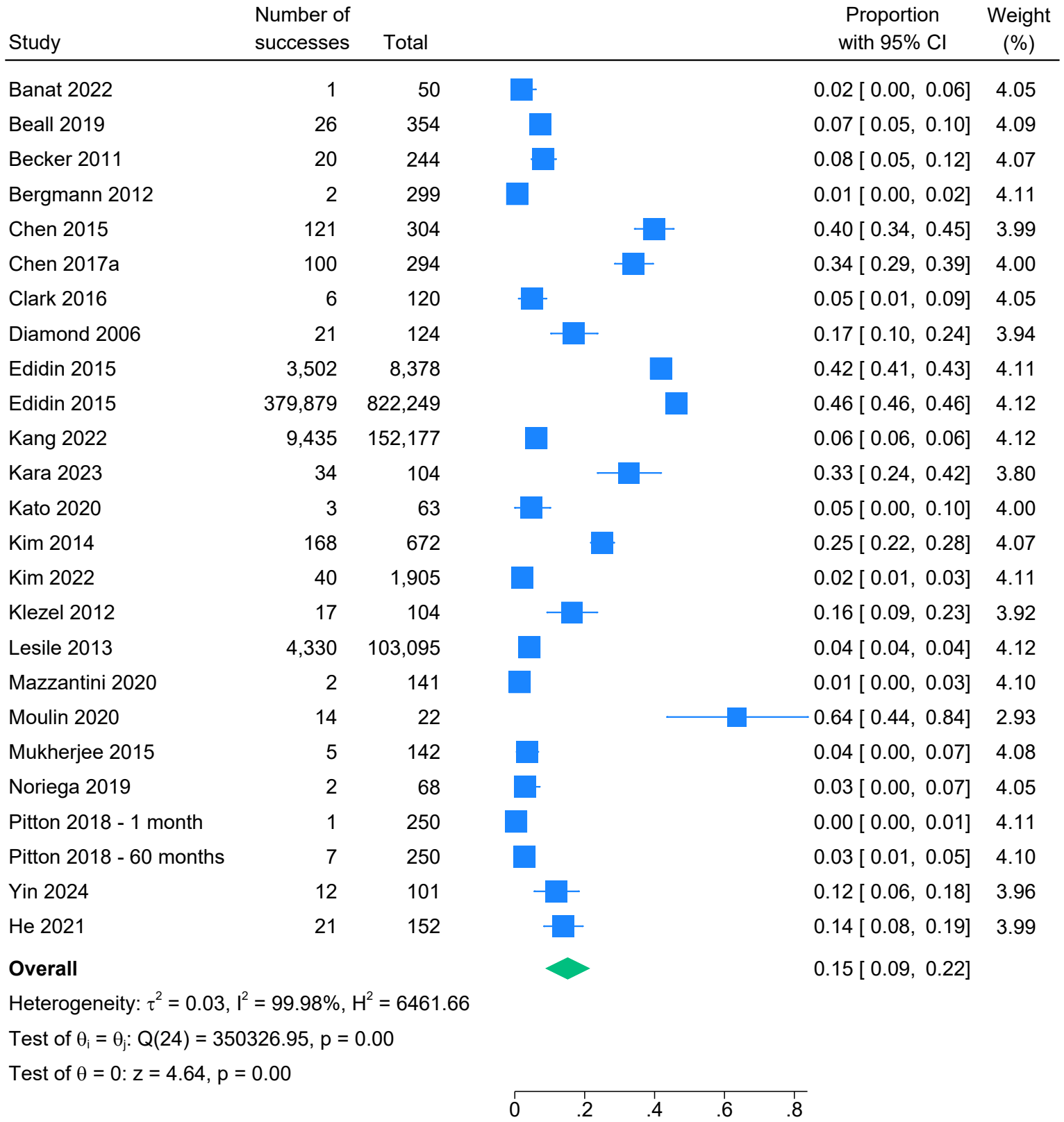

Random-effects REML model

Supplement: Supplementary file 1 [file jcm-14-08230-s001.zip › Figure S6.pdf]

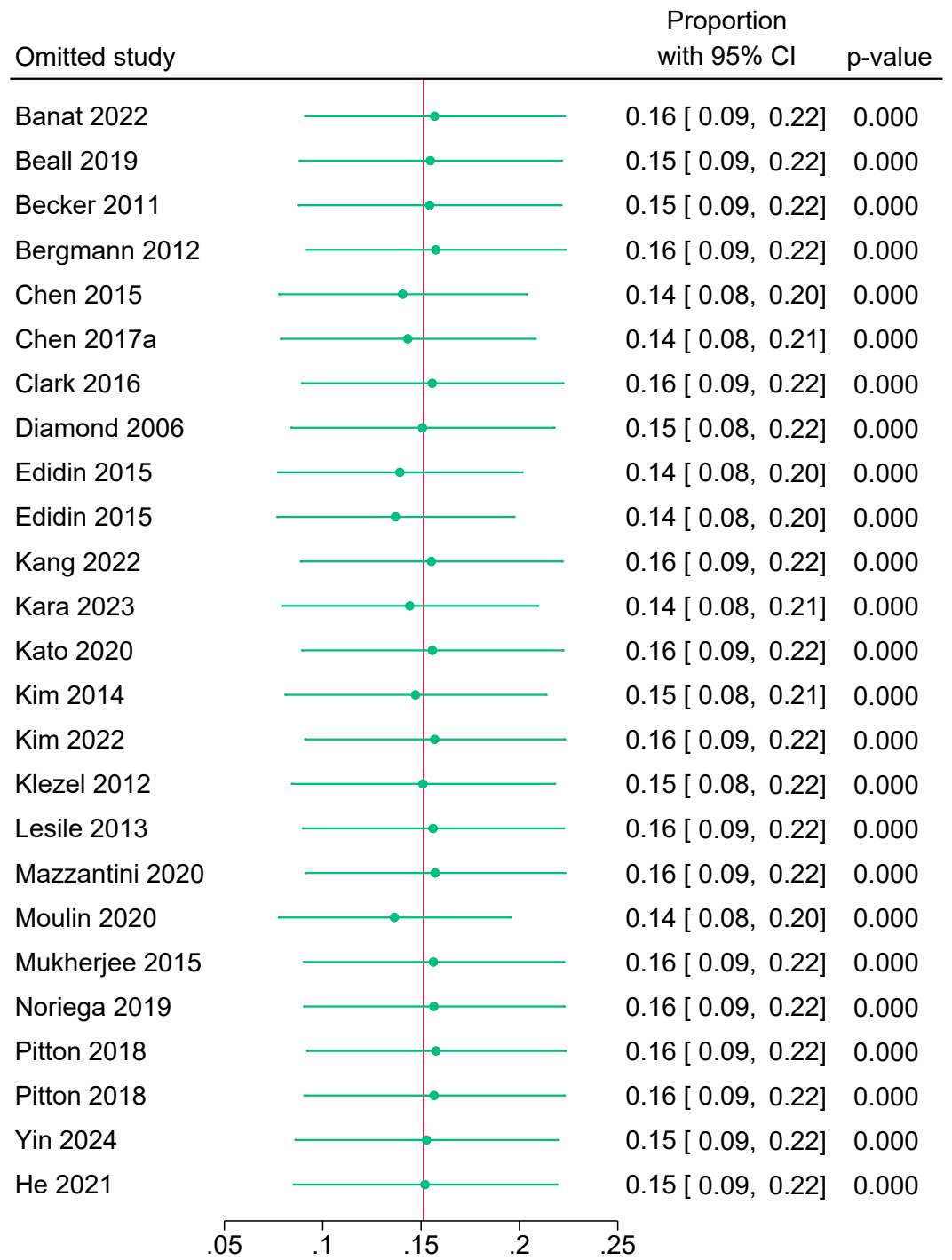

Random-effects REML model

Supplement: Supplementary file 1 [file jcm-14-08230-s001.zip › Figure S7.pdf]

# Galbraith plot

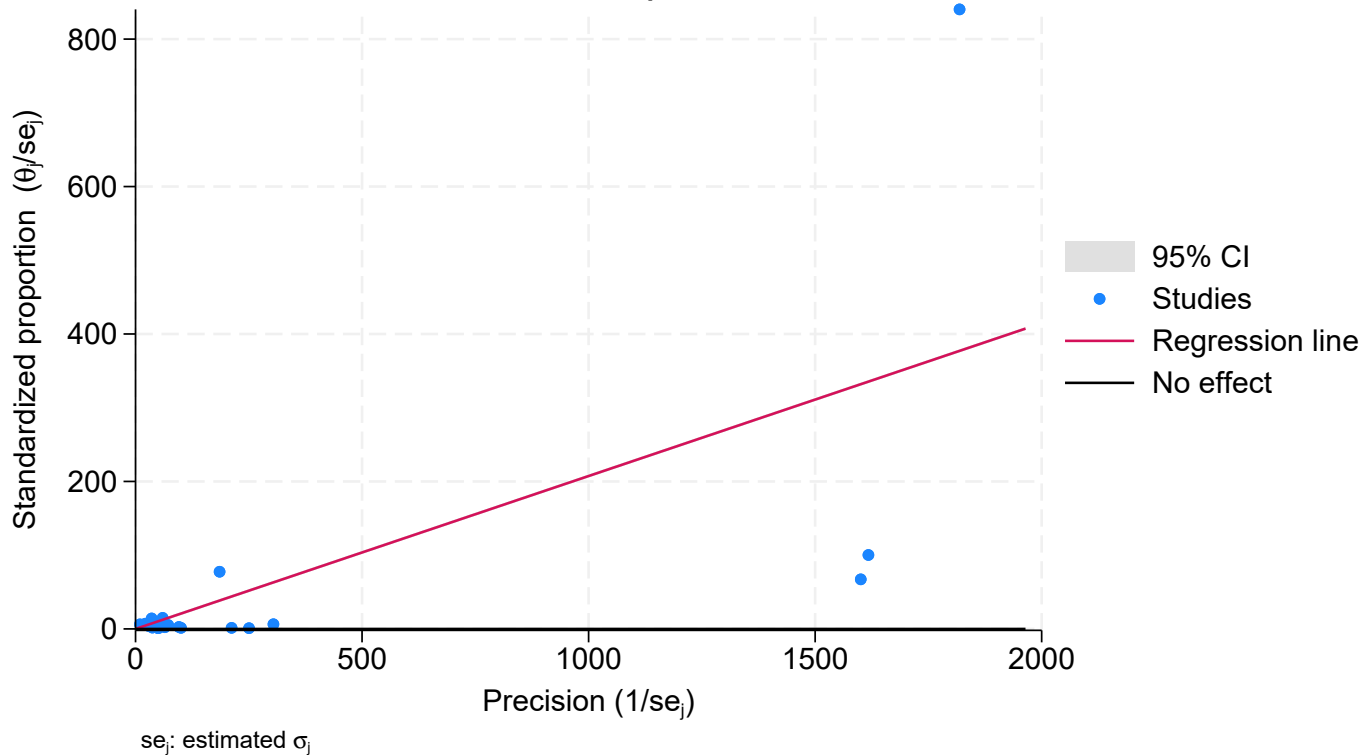

Supplement: Supplementary file 1 [file jcm-14-08230-s001.zip › Figure S8.pdf]

Funnel plot

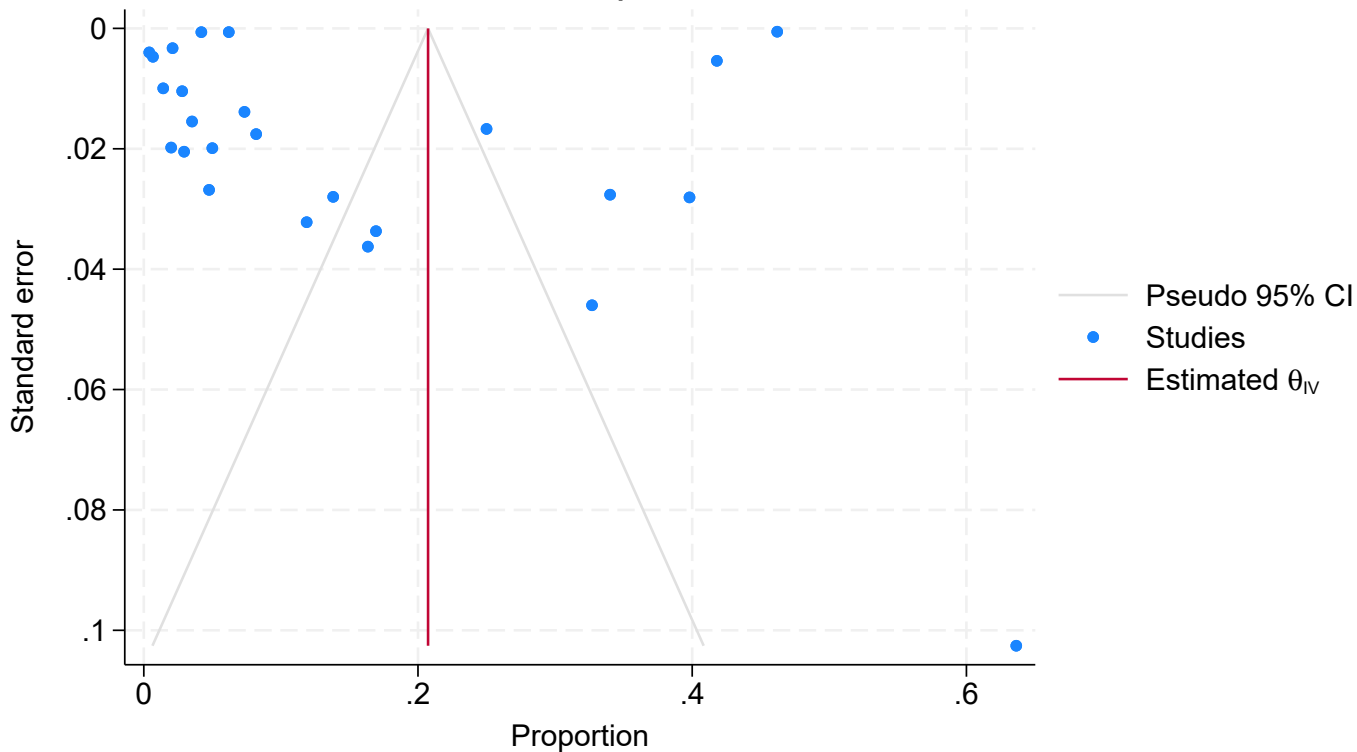

Supplement: Supplementary file 1 [file jcm-14-08230-s001.zip › Figure S9.pdf]
